# SGLT2 inhibitors and diabetic retinopathy progression: evidence from a retrospective cohort study and Mendelian randomization analysis

**DOI:** 10.3389/fendo.2026.1846454

**Published:** 2026-07-16

**Authors:** Mengya Wang, Tianwei Liu, Bojun Zhao

**Affiliations:** 1 Department of Ophthalmology, Shandong Provincial Hospital, Shandong University, Jinan, Shandong, China; 2 Department of Ophthalmology, Shandong Second Provincial General Hospital, Jinan, Shandong, China; 3 Department of Ophthalmology, The Second People’s Hospital of Jinan, Jinan, Shandong, China

**Keywords:** diabetic retinopathy, Mendelian randomization, microvascular complications of diabetes, optical coherence tomography angiography (OCTA), SGLT2 (sodium-glucose cotransporter 2) inhibitor

## Abstract

**Aims:**

To investigate the association between sodium-glucose cotransporter 2 inhibitors (SGLT2i) use and diabetic retinopathy (DR) progression, and to explore supporting genetic and molecular evidence using a retrospective cohort study with Mendelian randomization (MR).

**Materials and methods:**

In this retrospective cohort study at Shandong Provincial Hospital from January 2023 to December 2024, patients with type 2 diabetes on stable SGLT2i plus basal insulin (SGLT2i+INS) or sulfonylureas plus basal insulin (SUL+INS) were included. Wide-field swept-source optical coherence tomography angiography (SS-OCTA) was used to assess retinal microvascular parameters. Kaplan–Meier and Cox regression analyses evaluated DR progression and new-onset diabetic macular edema with sensitivity analyses performed to assess robustness. An exploratory nomogram was developed and evaluated by decision curve analysis. Two-sample MR and two-step mediation analyses were conducted to examine the genetic association between SGLT2 and DR and to identify potential mediating metabolites and plasma proteins.

**Results:**

A total of 191 eyes were included: 56 in the SGLT2i+INS group and 135 in the SUL+INS group. SGLT2i+INS was associated with a lower risk of DR progression (HR = 0.40, 95% CI 0.19–0.84; P = 0.016) and reduced cumulative incidence (log-rank P = 0.032). The exploratory OCTA-based nomogram showed modest performance (C-index=0.705). MR supported an association between genetically proxied SGLT2 and DR risk (OR = 1.21, 95% CI 1.05–1.39; P = 0.009). Nine metabolites and five plasma proteins showed nominal evidence of potential mediation.

**Conclusions:**

Among insulin-treated patients with type 2 diabetes, SGLT2i use was associated with a lower risk of DR progression than sulfonylurea use. OCTA parameters and candidate molecular mediators may provide exploratory prognostic and biological insights, warranting validation in larger prospective studies and randomized trials.

## 1 Introduction

Diabetes mellitus (DM) affects nearly half a billion people globally, posing a substantial public health challenge (1). Diabetic retinopathy (DR) affects about 22% of patients with diabetes, and its burden is expected to increase further with the rising prevalence of DM and longer patient survival (2). Although advances in diabetes management have reduced macrovascular mortality, improved survival has increased the burden of vision-threatening microvascular complications such as DR (3). These trends underscore the need for pharmacological strategies targeting DR.

Although optimal control of glycemia, serum lipids, and blood pressure can reduce the risk of microvascular complications (4), pharmacological strategies specifically targeting diabetic microangiopathy remain limited (5). Originally designed to enhance glycemic control by blocking renal glucose reabsorption, sodium-glucose cotransporter 2 inhibitors (SGLT2i) are now recommended for cardiorenal protection in combination therapy for type 2 diabetes mellitus (T2DM) (6). Notably, the expression of SGLT2 in the human retina suggests that it may represent a therapeutic target for DR (7). Preclinical studies demonstrate that SGLT2i confer retinoprotection through mechanisms beyond glycemic control, including attenuation of oxidative stress, inflammation, and blood-retinal barrier disruption (8, 9). Nevertheless, clinical evidence in T2DM is still mixed. Some studies suggest neutral ocular effects (10), whereas others indicate that SGLT2i may slow DR progression or reduce diabetic fundus disease, particularly in combination with insulin therapy (11, 12). Owing to residual confounding, heterogeneous endpoint definitions, and variable ophthalmic surveillance, the association between SGLT2i use and DR progression remains uncertain.

Swept−source optical coherence tomography angiography (SS-OCTA) enables noninvasive, three-dimensional visualization and quantitative assessment of the retinal microvasculature, making it increasingly valuable for posterior segment diseases (13). It can detect subclinical microvascular alterations, including changes in the foveal avascular zone and reductions in vessel density (VD), before overt clinical manifestations of DR (14, 15). OCTA-derived parameters may therefore serve as objective biomarkers for retinal microvascular status and progression in DR and diabetic macular edema (DME) (16–18).

In this retrospective cohort study, we evaluated the association between SGLT2i combined with insulin therapy and DR progression, with new-onset DME assessed as an additional outcome. We also explored the prognostic relevance of baseline OCTA-derived microvascular parameters for DR progression risk. In addition, Mendelian randomization (MR) and mediation analyses were performed to provide complementary genetic evidence and explore potential mediating pathways linking SGLT2 to DR.

## 2 Methods

### 2.1 Participants

**Figure 1:**
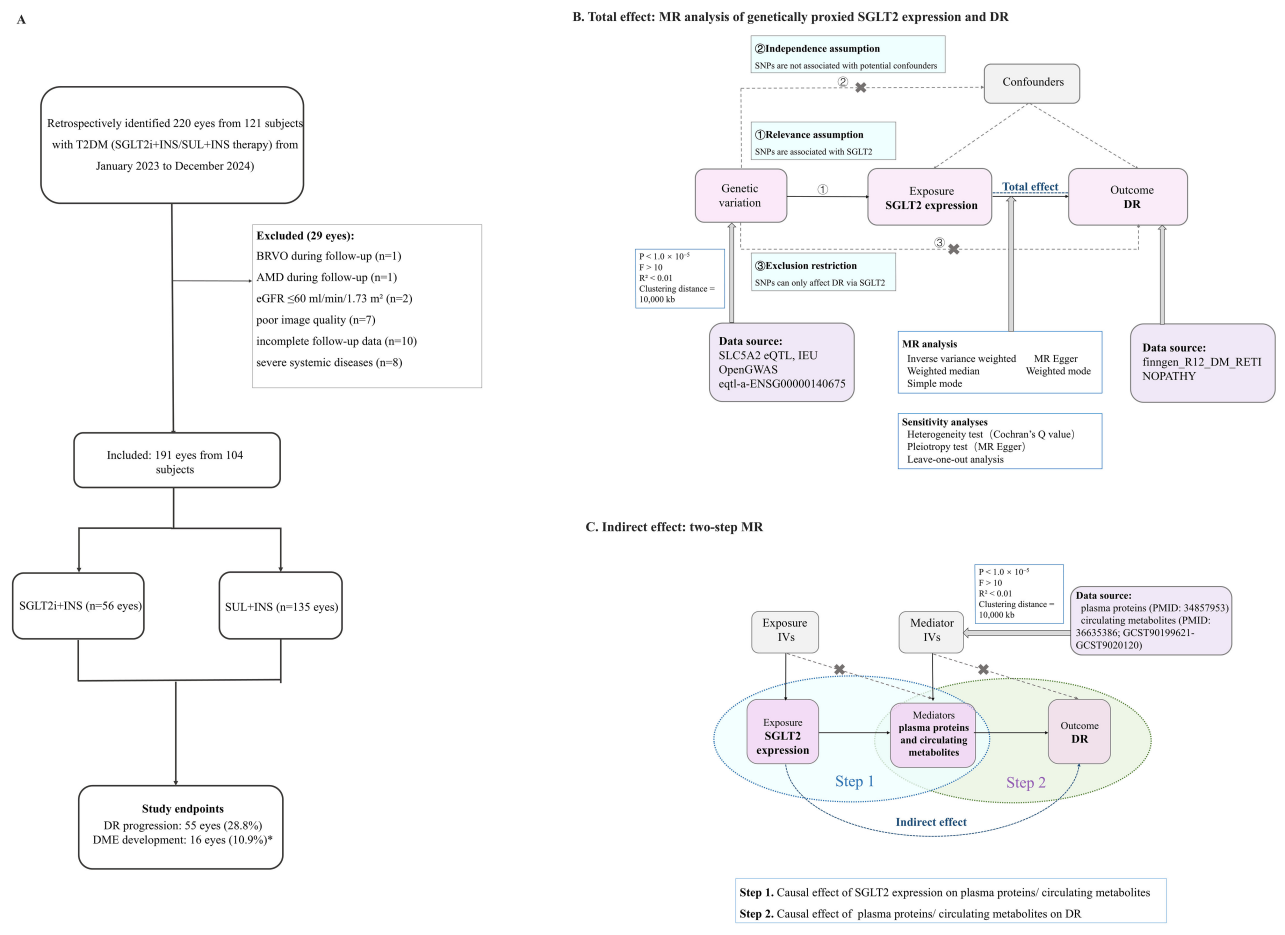
Study flowchart of the retrospective cohort and MR framework. **(A)** Retrospective cohort selection process. Of 220 eyes (121 patients) with T2DM receiving SGLT2i+INS or SUL+INS, 191 eyes (104 patients) were included after exclusion. Study endpoints were DR progression and DME development. *For DME analysis, 44 eyes with baseline DME were further excluded, leaving 147 eyes. **(B)** Total-effect MR framework evaluating the causal effect of genetically proxied SGLT2 expression on DR under the three core MR assumptions. Genetic instruments were obtained from SLC5A2 eQTL data, and DR summary statistics were obtained from GWAS datasets. **(C)** Two-step MR framework assessing indirect effects through plasma proteins and circulating metabolites: step 1, genetically proxied SGLT2 expression to candidate mediators; step 2, candidate mediators to DR. AMD, age-related macular degeneration; BRVO, branch retinal vein occlusion; DME, diabetic macular edema; DR, diabetic retinopathy; eGFR, estimated glomerular filtration rate; eQTL, expression quantitative trait locus; GWAS, genome-wide association study; INS, insulin; MR, Mendelian randomization; SGLT2, sodium-glucose cotransporter 2; SGLT2i, sodium-glucose cotransporter 2 inhibitor; SUL, sulfonylurea; T2DM, type 2 diabetes mellitus.

This retrospective cohort study adhered to the STROBE reporting guidelines. The Institutional Review Board of Shandong Provincial Hospital approved the study protocol (SWYX: NO.2024-028) in January 2024, including retrospective review of data from January 2023 onward, in accordance with the Declaration of Helsinki. Consecutive patients with diabetes who underwent ophthalmic examination at Shandong Provincial Hospital between January 2023 and December 2024 were identified from electronic medical records, with follow-up data collected until June 30, 2025. All examinations and treatments were part of routine clinical care. Given the retrospective design and use of de-identified data, a waiver of informed consent was granted. The participant enrollment and exclusion process is illustrated in **Figure 1A**.

Inclusion criteria included:(1) age ≥18 years; (2) T2DM diagnosed according to the 2024 American Diabetes Association (ADA) criteria; (3) stable hypoglycemic therapy for at least three months before enrollment, limited to SGLT2i with basal insulin (SGLT2i+INS) or sulfonylurea with basal insulin (SUL+INS), with basal insulin defined as once-daily long-acting insulin analogs used for stable fasting blood glucose; and (4) complete baseline and follow-up data. Exclusion criteria included: (1) other ocular diseases at baseline or during follow-ups, such as glaucoma, retinal vein occlusion, or age-related macular degeneration; (2) history of retinal laser photocoagulation, vitrectomy, or anti-vascular endothelial growth factor (VEGF) therapy within 6 months before enrollment; (3) estimated glomerular filtration rate(eGFR) ≤ 60mL/min/1.73m^2^ at baseline; (4) incomplete follow-up data or loss to follow-up within 6 months after enrollment; (5) severe systemic diseases, including myocardial infarction, end-stage renal disease, or malignant neoplasms; (6) poor-quality imaging data; and (7) cataract surgery within 6 months before DME onset in the incident DME analysis, to exclude Irvine-Gass syndrome.

All subjects underwent best corrected visual acuity (BCVA) assessment, reported as logarithm of the minimum angle of resolution (LogMAR). DR severity was assessed by dilated fundus examination using the modified Airlie House ETDRS criteria, classifying it into mild, moderate, or severe non-proliferative diabetic retinopathy (NPDR), or proliferative DR (PDR) (19).

### 2.2 SS-OCT/OCTA examination

All subjects underwent wide-field SS-OCTA using the VG100D system (SVision Imaging, Ltd., China), from which structural optical coherence tomography (OCT) and OCTA parameters were obtained. A 15 × 12 mm fovea-centered macular scan was acquired at a wavelength of 1050 nm, with an acquisition speed of 100,000 A-scans per second an axial optical resolution of 3.8 μm, and an axial digital resolution of 2.0 μm.

Quantitative analysis evaluated macular retinal thickness (MRT), thicknesses of the retinal nerve fiber layer (RNFL), ganglion cell layer and inner plexiform layer (GCL+IPL), ganglion cell complex (GCC), and choroid, as well as VD and perfusion area (PA) across the superficial, intermediate, deep, and choriocapillary plexus. Choroidal vascular index (CVI) and choroidal vascular volume (CVV) were also quantified. Layer definitions are provided in **Supplementary Table S1**. These parameters were quantified in four concentric regions: central fovea (3 mm diameter), perifoveal (3–6 mm), pararetinal (6–9 mm), and periretinal (9–12 mm) rings (**Supplementary Figure 1**). For baseline group comparisons and Cox regression analyses, only the macula-centered 6 × 6 mm OCT/OCTA metrics were used to ensure consistency with **Table 1** and the main prognostic models.

**Table 1:** Baseline characteristics of patients treated with SGLT2i+INS or SUL+INS.

Characteristics	SGLT2i+INS (N = 56)		SUL+INS (N = 135)		P value
	N	Mean (SD) or %	N	Mean (SD) or %	
Age (year)	56	56.80 (2.00)	135	59.86 (1.17)	0.185
Sex					0.148
Female	13	23.2	51	37.8	
Male	43	76.8	84	62.2	
Duration of diabetes (year)	56	13.95 (1.44)	134	14.23 (0.75)	0.860
Body mass index (kg/m²)	56	25.44 (0.46)	130	25.52 (0.46)	0.891
logMAR	56	0.37 (0.06)	135	0.46 (0.03)	0.268
Severity of DR					0.659
Moderate NPDR	21	37.5	38	28.1	
Severe NPDR	27	48.2	85	63.0	
PDR	8	14.3	12	8.9	
Baseline DME (yes)	8	14.3	36	26.7	0.153
HbA1c (%)	56	8.56 (0.22)	124	8.82 (0.17)	0.333
Comorbidities (yes)					
Hypertension	27	51.9	77	63.6	0.289
Hyperlipidemia	30	53.6	67	51.5	0.851
DN	30	53.6	54	41.5	0.265
Smoke					0.308
Never	33	61.1	82	72.6	
Former	9	16.7	12	10.6	
Current	12	22.2	19	16.8	
SS-OCT/OCTA Metrics a					
Superficial Capillary Plexus					
Vessel density (%)	56	45.93 (0.77)	135	43.54 (0.80)	**0.031**
Perfusion area (mm²)	56	11.69 (0.19)	135	10.95 (0.21)	**0.010**
Deep Capillary Plexus					
Vessel density (%)	56	6.29 (0.67)	135	4.43 (0.45)	**0.021**
Perfusion area (mm²)	56	5.75 (0.19)	135	4.71 (0.17)	**0.001**
Macular retinal thickness (μm)	56	320.11 (4.43)	135	314.03 (3.96)	0.306

^a^ SS-OCT/OCTA metrics were derived only from the macula-centered 6 × 6-mm field to facilitate inter-group quality control. Group sample sizes are shown in the column headers; variable-specific denominators differ because of missing baseline data.

DME, diabetic macular edema; DN, diabetic nephropathy; DR, diabetic retinopathy; HbA1c, glycated hemoglobin; INS, insulin; logMAR, logarithm of the minimum angle of resolution; NPDR, non-proliferative diabetic retinopathy; PDR, proliferative diabetic retinopathy; SD, standard deviation; SGLT2i, sodium-glucose cotransporter 2 inhibitor; SS-OCTA, swept-source optical coherence tomography angiography; SUL, sulfonylurea.

The bold values indicate statistically significant results, defined as p < 0.05.

Analyses were performed using software version 3.1.255 (VG100D; SVision Imaging, Ltd., China). Automated segmentation and quantification were reviewed and manually corrected when necessary. Two licensed retinal specialists independently reviewed the images and extracted the measurements using the built-in software.

### 2.3 Definitions of end points

Primary endpoint: DR progression. A single 200° wide-field digital fundus photograph (fovea-centered) was obtained per eye per visit using a nonmydriatic camera (P200T, OPTOS PLC, Fife, KY11 8GR, UK). DR severity was assessed at baseline and follow-up by a fundus disease retinal specialist (Mengya Wang), who was experienced in DR grading and masked to treatment group, using the modified Airlie House classification (15-step scale, 10–65: no DR to severe proliferative DR) (20). DR progression was characterized by a ≥2-step increase from baseline, applicable to eyes with initial grades ranging from 10 to 65 (21).

Secondary endpoint: DME development defined as center-involved macular edema at follow-up with a central subfield thickness of ≥305 μm in men or ≥290 μm in women (22). Eyes with baseline DME were retained for DR progression analysis but excluded from incident DME analysis (16).

### 2.4 Assessment of covariates

All clinical and OCTA covariates included in the analyses were assessed at study inclusion. Age was recorded at baseline. All participants were documented as Han Chinese in the medical records; therefore, ethnicity was not included as a covariate. Diabetes duration was defined as the interval between the initial diagnosis and the baseline visit. The glycated hemoglobin (HbA1c) level was obtained from the most recent laboratory records. Body mass index (BMI) was calculated as weight divided by height squared. Smoking status, based on self-report, was categorized as never, former, or current. Hypertension was defined as systolic blood pressure ≥130 mmHg, diastolic blood pressure ≥80 mmHg, use of antihypertensive medication, or a self-reported history. Hyperlipidemia was defined as total cholesterol ≥6.2 mmol/L, triglycerides ≥1.7 mmol/L, low-density lipoprotein cholesterol (LDL-C) ≥3.4 mmol/L, high-density lipoprotein cholesterol (HDL-C) <1.0 mmol/L, current antihyperlipidemic medication use, or a self-reported history. Diabetic nephropathy (DN) was identified from medical records after exclusion of other primary kidney diseases. Because participants with estimated glomerular filtration rate (eGFR) ≤60 mL/min/1.73m² were excluded, DN mainly represented diabetic kidney involvement with preserved renal function. Superficial capillary plexus (SCP) and deep capillary plexus (DCP) VD were included as OCTA covariates in the fully adjusted Cox model.

### 2.5 Sensitivity analysis

Sensitivity analyses were conducted within a multiple-imputation framework using five imputed datasets to assess the robustness of the primary findings. Multiple imputation was performed for variables included in the Cox regression models. First, cluster-robust standard errors were applied to account for inter-eye correlation, with clustering at the patient level. Second, a single-eye analysis was performed with 100 iterations per imputed dataset by randomly selecting one eye per patient. Third, a simplified model excluding SCP VD was evaluated. Fourth, inverse probability of treatment weighting (IPTW) was used to reduce treatment-selection bias. The propensity score model included all covariates in the fully adjusted Cox model. Stabilized weights were calculated using the marginal treatment prevalence, and extreme weights were truncated at the 99th percentile. Estimates from the five imputed datasets were pooled using Rubin’s rules, and compared using hazard ratios (HR), 95% confidence intervals (95% CI), and C-index values. All sensitivity analyses were conducted using R version 4.5.2 (R Foundation for Statistical Computing, Vienna, Austria).

### 2.6 MR analysis

Following STROBE-MR guidelines, a two-sample MR analysis was performed to explore the association between genetically proxied SGLT2 expression and DR (**Figure 1B**). Two-step MR mediation analysis further explored potential mediating roles of 1,400 circulating metabolites and 47 plasma proteins (**Figure 1C**). Expression quantitative trait locus (eQTL) data for SLC5A2, encoding SGLT2, were sourced from the IEU Open Genome-Wide Association Studies (GWAS) database (23). Instrumental variables for SGLT2 expression were selected with P < 1×10^−5^, linkage disequilibrium r² < 0.01, and F-statistic >10 (24). DR GWAS summary data, comprising 13,167 cases and 131,272 controls, were obtained from the FinnGen consortium. Dataset details are provided in **Supplementary Table S2**. SNP effect alleles and beta coefficients were harmonized across the exposure, mediator, and outcome datasets. The primary causal estimates were calculated with the inverse variance-weighted (IVW) method. Potential mediation was assessed using the product-of-coefficients method, with Benjamini-Hochberg FDR correction and delta-method confidence intervals (25). Sensitivity analyses included Cochran’s Q test for heterogeneity, MR-Egger regression for pleiotropy, and leave-one-out analysis to evaluate single-SNP influence.

### 2.7 Statistical analysis

Each eligible eye was treated as the analytical unit. Baseline group differences and OCT/OCTA differences according to baseline DME status were assessed using generalized estimating equation (GEE) models. Continuous variables are expressed as mean ± standard deviation (SD), and categorical variables as number (percentage).

Kaplan-Meier analysis and the log-rank test were used to compare the cumulative incidence of DR progression and DME development between the SGLT2i+INS and SUL+INS groups. Cox proportional hazards models were fitted using the R package “survival” to examine the associations of SGLT2i use with study outcomes. Variables significant in univariate analysis were assessed for multicollinearity using variance inflation factors (VIF) and for the proportional hazards (PH) assumption using Schoenfeld residuals before inclusion in multivariable models. MRT was summarized descriptively but excluded from Cox models because the primary endpoint was DR progression rather than DME, and inclusion of additional structural parameters could increase overfitting risk. SCP and DCP VD were converted into quartiles and entered into the Cox models as categorical variables, with the highest VD quartile used as the reference group.

We constructed three sequential models: Model 1 adjusted for treatment group, age, sex, and diabetes duration; Model 2 adjusted for these factors plus HbA1c, baseline DR severity, and DN; and Model 3 adjusted for all covariates. Model fit, discrimination, and parsimony were evaluated using the likelihood ratio test, C-index with delta C-index (ΔC), and Akaike Information Criterion (AIC), respectively (26). The ΔC-index was calculated relative to Model 1. The final exploratory model was internally validated using bootstrap resampling, with optimism-corrected performance reported. An exploratory nomogram and decision curve analysis (DCA) based on Model 3 were presented in the **Supplementary Materials**. Statistical analyses were conducted using R version 4.5.2 (R Foundation for Statistical Computing, Vienna, Austria) and SPSS version 26.0 (IBM Corp., Armonk, NY, USA). A two-tailed P-value below 0.05 was deemed statistically significant.

## 3 Results

### 3.1 Baseline characteristics of the study population

**Figure 1A** shows that 220 eligible eyes were initially screened, with 191 eyes included in the final cohort after exclusions. Baseline characteristics were balanced between the included and excluded subjects (**Supplementary Table S3**). As shown in **Table 1**, 56 eyes were included in the SGLT2i+INS group and 135 in the SUL+INS group. The enrolled participants had a mean age of 58.99 ± 1.04 years and a mean diabetes duration of 13.96 ± 0.78 years. No statistically significant between-group differences were observed in demographic characteristics, metabolic profiles, baseline DR severity, baseline DME prevalence, macula-centered 6×6 mm MRT, or major comorbidities (all P > 0.05). At baseline, DME was present in 8 of 56 eyes (14.3%) in the SGLT2i+INS group and 36 of 135 eyes (26.7%) in the SUL+INS group, with no statistically significant between-group difference (P = 0.153). Over a median follow-up of 17.7 months (interquartile range [IQR], 13.14–21.1), 55 of 191 eyes (28.8%) experienced DR progression.

Among the 191 eyes, 44 had baseline DME and were therefore included in the DR progression analysis but excluded from the analysis of incident DME. Of the 147 eyes without baseline DME, 16 (10.9%) developed DME during follow-up. Baseline structural OCT and OCTA parameters stratified by DME status are presented descriptively in **Supplementary Tables S4**–**S7** and **Supplementary Figure 2**. Compared with eyes without baseline DME, eyes with baseline DME showed significantly lower DCP-related VD and PA, lower CVV and CVI, and thinner GCC, RNFL, and choroid, particularly in the superior and temporal quadrants (all P < 0.05). Because only 16 incident DME events occurred, these DME-related comparisons were considered descriptive and exploratory.

### 3.2 Survival analysis

**Figure 2:**
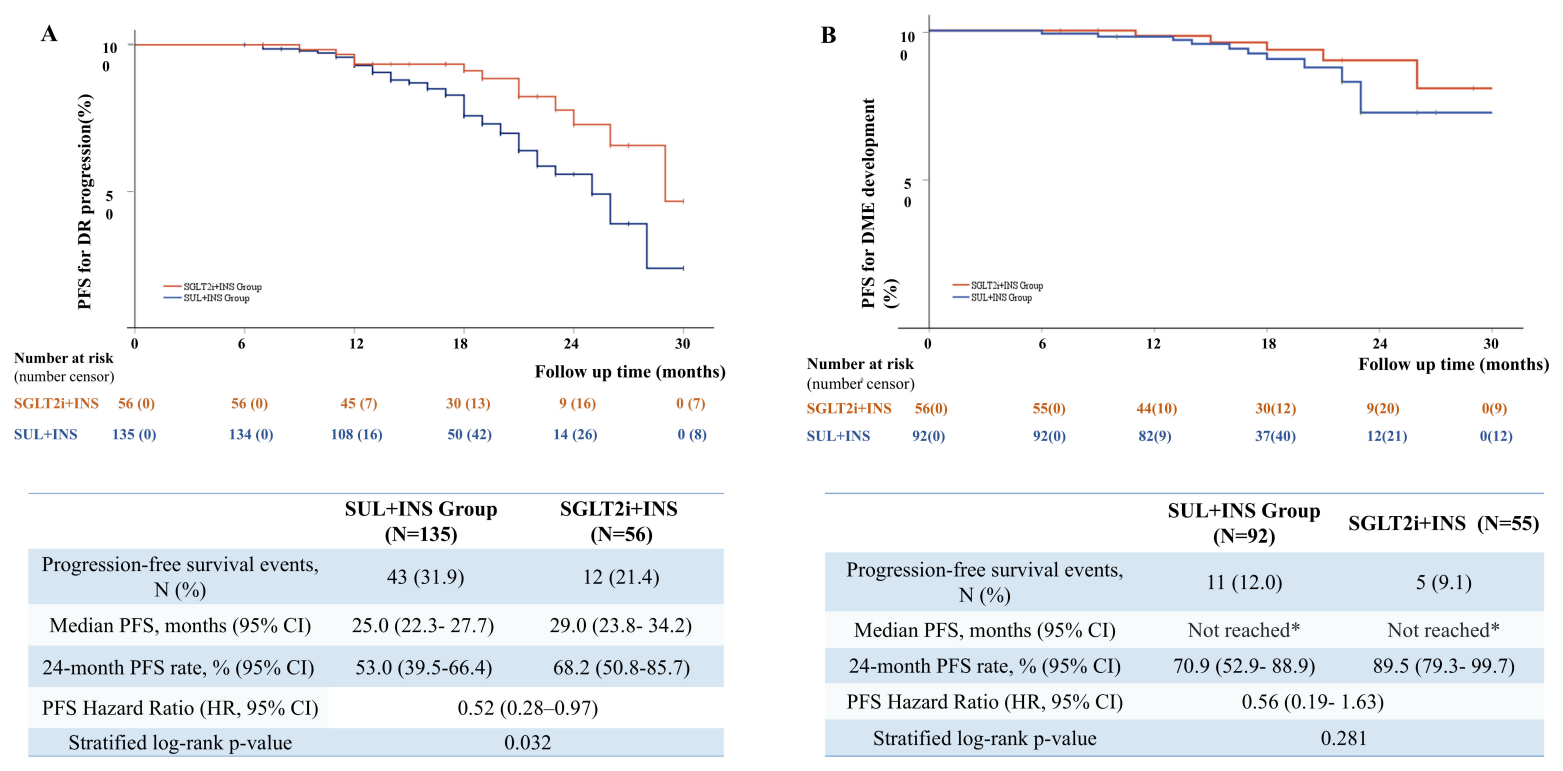
Kaplan–Meier curves of progression-free survival for DR progression and DME development in the SGLT2i+INS and SUL+INS groups. **(A)** Kaplan–Meier curve showing progression-free survival for DR progression in eyes treated with SGLT2i+INS or SUL+INS. The numbers at risk and censored observations are shown below the curves. Eyes in the SGLT2i+INS group showed longer progression-free survival than those in the SUL+INS group, with a stratified log-rank p-value of 0.032. The summary table below panel A shows the progression-free survival metrics for DR progression, including the number of events, median PFS, 24-month PFS rate, hazard ratio, and stratified log-rank p-value. **(B)** Kaplan–Meier curve showing progression-free survival for DME development in eyes treated with SGLT2i+INS or SUL+INS. The numbers at risk and censored observations are shown below the curves. No significant difference in progression-free survival was observed between the two groups, with a stratified log-rank p-value of 0.281. The summary table below panel B shows the progression-free survival metrics for DME development, including the number of events, median PFS, 24-month PFS rate, hazard ratio, and stratified log-rank p-value. Abbreviations: CI, confidence interval; DME, diabetic macular edema; DR, diabetic retinopathy; HR, hazard ratio; INS, insulin; PFS, progression-free survival; SGLT2i, sodium-glucose cotransporter 2 inhibitor; SUL, sulfonylurea.

The primary study outcome was progression of DR. During the observation period, this event was recorded in 12 of 56 eyes receiving SGLT2i+INS and in 43 of 135 eyes receiving SUL+INS (**Figure 2A**). Estimated cumulative event rates were 21.4% and 31.9%, respectively. Kaplan–Meier analysis showed better DR progression-free survival in the SGLT2i+INS group (log-rank P = 0.032; **Figure 2A**).

The secondary outcome was incident DME. Among eyes without DME at baseline, 5 of 55 eyes in the SGLT2i+INS group and 11 of 92 eyes in the SUL+INS group developed DME over time. The estimated cumulative incidence was 9.1% for SGLT2i+INS and 12.0% for SUL+INS. Although incidence was numerically lower in the SGLT2i+INS group, the between-group difference was not significant (log-rank P = 0.281; **Figure 2B**).

### 3.3 Cox proportional hazards models: univariate and multivariate approaches

**Supplementary Table S8** presents the univariate associations of all factors with DR and DME development. Variables significantly associated with ≥2-step DR progression and meeting multicollinearity and proportional hazards assumption (**Supplementary Tables S9**, **S10**) were entered into multivariable Cox models, including treatment group, baseline DR severity, HbA1c, DN, SCP VD, and DCP VD.

Compared with SUL+INS, SGLT2i+INS was associated with a lower risk of DR progression in Model 1 (HR = 0.47, 95% CI: 0.25–0.90, P = 0.022; **Table 2**) and Model 2 (HR = 0.40, 95% CI: 0.20–0.82, P = 0.012; **Table 2**). In the fully adjusted model, SGLT2i+INS remained significantly associated with reduced DR progression risk (HR = 0.40, 95% CI: 0.19–0.84, P = 0.016; **Table 2**).

**Table 2:** Multivariable Cox regression analysis for DR progression.

Variables	Model 1^a^	P value	Model 2^b^	P value	Model 3^c^	P value
	HR (95% CI)		HR (95% CI)		HR (95% CI)	
Treatment Group (SUL+INS)	Ref.	–	Ref.	–	Ref.	–
SGLT2i + INS	0.47 (0.25-0.90)	**0.022**	0.40 (0.20-0.82)	**0.012**	0.40 (0.19-0.84)	**0.016**
Age (per year)	0.99 (0.96-1.02)	0.609	1.01 (0.97-1.04)	0.679	1.01 (0.97-1.04)	
Sex (female)	Ref.	–	Ref.	–	Ref.	0.089
Male	1.34 (0.72-2.47)	0.348	1.97 (1.00-3.88)	0.051	1.87 (0.91-3.83)	0.770
Duration of diabetes (per year)	1.03 (0.99-1.07)	0.163	1.00 (0.96-1.05)	0.916	1.01 (0.96-1.07)	0.649
Severity of DR (Moderate NPDR)	–	–	Ref.	–	Ref.	
Severe NPDR	–	–	1.02 (0.44-2.37)	0.962	1.07 (0.44-2.61)	0.878
PDR	–	–	1.83 (0.66-5.03)	0.246	3.13 (1.00-9.80)	0.050
HbA1c (%)	–	–	1.18 (0.95-1.48)	0.139	1.09 (0.87-1.37)	0.444
DN (No)			Ref.	–	Ref.	
Yes	–	–	2.31 (1.16-4.63)	**0.018**	2.03 (0.99-4.15)	0.054
VD of SCP						
Q1 (highest)	–	–	Ref.	–	Ref.	
Q2	–	–	–	–	1.36 (0.5-3.68)	0.543
Q3	–	–	–	–	1.51 (0.56-4.11)	0.419
Q4 (lowest)	–	–	–	–	2.07 (0.79-5.44)	0.141
VD of DCP						
Q1 (highest)	–	–	–	–	Ref.	
Q2	–	–	–	–	2.11 (0.73-6.06)	0.166
Q3	–	–	–	–	2.77 (1.02-7.49)	**0.045**
Q4 (lowest)	–	–	–	–	2.65 (1.00-7.02)	**0.050**

^a^ Model 1 was adjusted for treatment group, age, sex, and duration of diabetes.

^b^ Model 2 was additionally adjusted for HbA1c, baseline DR severity, and diabetic nephropathy.

^c^ Model 3 was additionally adjusted for VD of SCP and DCP (categorized into quartiles). For SCP VD and DCP VD, quartiles were coded in descending order, with Q1 indicating the highest vessel density and Q4 the lowest.

CI, confidence interval; DCP, deep capillary plexus; DN, diabetic nephropathy; DR, diabetic retinopathy; HbA1c, glycated hemoglobin; HR, hazard ratio; Ref., reference group; SCP, superficial capillary plexus; VD, vessel density.

The bold values indicate statistically significant results, defined as p < 0.05.

DCP VD was modeled in quartiles, with Q1 representing the highest vessel density and used as the reference group. Relative to Q1, eyes in Q3 showed a significantly higher risk of DR progression (HR = 2.77, 95% CI: 1.02–7.49, P = 0.045), and eyes in Q4 showed a borderline association (HR = 2.65, 95% CI: 1.00–7.02, P = 0.050), suggesting that lower DCP VD may be associated with increased progression risk. No significant association was observed between treatment group and incident DME (HR = 0.56, 95% CI: 0.19–1.63, P = 0.288; **Supplementary Table S8**).

### 3.4 Sensitivity analyses

Using five multiply-imputed datasets (**Supplementary Table S11**), missing data for DN (2.62%) and HbA1c (5.76%) we imputed. Four sensitivity analyses were performed, and all yielded HRs below 1, consistent with the primary finding. The cluster−robust and single−eye analyses showed borderline associations (HR = 0.38, 95% CI: 0.15–1.01, P = 0.053; and HR = 0.33, 95% CI: 0.18–1.06, P = 0.055, respectively). The simplified model remained statistically significant (HR = 0.42, 95% CI: 0.21–0.86, P = 0.017). IPTW, using the same covariates as the fully adjusted model, improved covariate balance overall, with only modest residual imbalance for DN (**Supplementary Table S12**), and yielded a significant protective association (HR = 0.48, 95% CI: 0.29–0.78, P = 0.003). Model discrimination remained stable overall, with C-index ranging from 0.666 to 0.706 (**Table 3**).

**Table 3:** Sensitivity analyses of the association between SGLT2i+INS use and DR progression.

Analysis	Model details	HR (95% CI)	P value	C-index
Primary analysis	Fully adjusted Cox model	0.40 (0.19 – 0.84)	0.016	0.705
Cluster-robust analysis	Fully adjusted Cox model with patient-level clustering	0.38 (0.15 – 1.01)	0.053	0.666
Single-eye analysis	Fully adjusted Cox model using one randomly selected eye per patient, repeated 100 times in each imputed dataset	0.33 (0.18 – 1.06)	0.055	0.706
Simplified model	Cox model excluding SCP vessel density	0.42 (0.21 – 0.86)	0.017	0.684
IPTW analysis	Weighted Cox model using stabilized IPTW	0.48 (0.29 – 0.78)	0.003	0.682

The reference group was SUL+INS. Sensitivity analyses were conducted using five multiply imputed datasets, and estimates were pooled using Rubin’s rules. Fully adjusted models included age, sex, diabetes duration, HbA1c, diabetic retinopathy severity, diabetic nephropathy, VD of SCP, and VD of DCP; the simplified model excluded VD of SCP. IPTW used stabilized weights truncated at the 99th percentile.

C-index, concordance index; CI, confidence interval; DCP, deep capillary plexus; DR, diabetic retinopathy; HbA1c, glycated hemoglobin; HR, hazard ratio; SCP, superficial capillary plexus; VD, vessel density.

### 3.5 Exploratory nomogram and decision curve analysis

**Supplementary Table S13** compares the predictive performance of the three Cox proportional hazards models. Predictive performance improved progressively from Model 1 to Model 3. Model 1 showed limited discrimination (C-index = 0.566, 95% CI: 0.468–0.664), whereas Model 2 showed improved discrimination (C-index = 0.651, 95% CI: 0.551–0.751). Model 3 achieved the highest discrimination (C-index = 0.705, 95% CI: 0.597–0.813), with a significant increase in C-index compared with Model 1 (ΔC-index = 0.162, P for ΔC-index = 0.001). Although Model 2 had a slightly lower AIC than Model 3, Model 3 provided the greatest discriminatory ability and incorporated the OCTA parameters of primary interest. Therefore, Model 3 was used to construct an exploratory nomogram based on variables measured at study inclusion for estimating 12- and 24-month DR progression-free survival (**Supplementary Figure 3**).

Bootstrap-based internal validation of Model 3 showed an optimism-corrected C-index of 0.623 and a calibration slope of 0.578, suggesting moderate discrimination after correction for optimism and indicating potential overfitting. The improved performance of Model 3 suggests that baseline OCTA-derived SCP and DCP VD may enhance prognostic risk stratification for DR progression. However, the non-monotonic point allocation for DCP VD across quartiles, imperfect calibration, and optimism-corrected validation results indicate that the nomogram should be interpreted cautiously and requires external validation (**Supplementary Figures 4A, B**). DCA further showed that Model 3 provided greater clinical net benefit than Models 1 and 2 across clinically relevant threshold probabilities (**Supplementary Figure 5A, B**). These analyses should therefore be regarded as exploratory prognostic assessments rather than evidence for mechanistic inference or direct clinical implementation.

### 3.6 MR analysis results

Nine independent SNPs served as genetic instruments for SGLT2, with F-statistics > 10, indicating adequate instrument strength. Using the IVW method, genetically proxied SLC5A2 expression, the gene encoding SGLT2, was associated with DR risk (OR = 1.21; 95% CI: 1.05–1.39; P = 8.92×10^−3^; **Supplementary Table S14**). No evidence of heterogeneity or directional horizontal pleiotropy was detected (**Supplementary Table S15**). Leave-one-out analyses did not suggest that the observed associations were driven by any single SNP.

In mediation analyses, nine metabolites and five plasma proteins showed nominally significant mediation effects in the SGLT2–DR pathway (uncorrected P < 0.05; **Supplementary Table S16**). No evidence of heterogeneity or directional horizontal pleiotropy for the exposure–mediator and mediator–outcome paths was detected (**Supplementary Table S17**). However, most associations did not remain significant after FDR correction (**Supplementary Table S16**); therefore, these mediation findings should be considered exploratory and require further validation.

## 4 Discussion

Our retrospective cohort analysis showed that SGLT2i combined with insulin was associated with a lower risk of DR progression, and this pattern remained stable in multiple sensitivity analyses, including cluster-robust, single-eye, simplified model, and IPTW approaches. This association persisted after adjustment for demographic factors, diabetes-related characteristics, baseline DR severity, DN, and OCTA-derived vascular parameters. In parallel, baseline OCTA-derived biomarkers were explored as complementary prognostic indicators beyond conventional clinical factors. The exploratory model showed improved discrimination after adding SCP and DCP VD, although calibration was imperfect and the model should not be considered ready for clinical risk stratification. At baseline, DME was more frequently observed in eyes with lower retinal and choroidal VD and PA, together with thinner GCC, RNFL, and choroid. However, SGLT2i plus insulin was not significantly associated with incident DME. MR and mediation analyses further provided exploratory evidence suggesting possible involvement of the SGLT2 pathway and related systemic metabolic or protein-level pathways in DR.

Existing clinical data regarding the impact of SGLT2i on DR remain inconclusive. Initial randomized trials, including EMPA-REG OUTCOME, indicated that empagliflozin did not elevate retinopathy risk compared to placebo (27). Likewise, a systematic review showed that SGLT2i use was not significantly associated with retinopathy events in T2DM (28). A network meta-analysis identified a potential drug-specific signal for canagliflozin, but not for empagliflozin or dapagliflozin (29). In contrast, real-world and simulation studies have suggested lower rates of sight-threatening retinopathy and DME with SGLT2i than with sulfonylureas, DPP-4 inhibitors, or pioglitazone (2, 11, 12).Our results are consistent with this growing real-world evidence and suggest that SGLT2i+INS therapy may be associated with a lower risk of DR progression than SUL+INS therapy. However, the absence of a significant association with new-onset DME should be interpreted cautiously, given the limited statistical power, relatively short follow-up, and multifactorial nature of DME.

Unlike earlier research primarily aimed at assessing the clinical association of SGLT2i in DR, our study additionally explored whether baseline OCTA-derived microvascular parameters could be incorporated into a risk prediction model for DR progression. Prior work has shown that OCTA parameters may provide incremental prognostic value beyond traditional risk factors and sensitively quantify DR-related microvascular changes (15, 16, 30). Regarding the treatment-associated findings, the association between SGLT2i+INS therapy and reduced DR progression appeared more evident in eyes with NPDR, particularly severe NPDR, but less apparent in PDR. This pattern should be interpreted cautiously because of the small number of PDR eyes, image-quality limitations in advanced retinopathy, and a possible ceiling effect once severe retinal damage is present. In the prediction analysis, the addition of OCTA-derived VD parameters improved model discrimination, and DCA suggested greater net benefit for the OCTA-enhanced model than for models based only on clinical variables. However, because calibration was imperfect and the risk pattern across DCP VD quartiles was non-monotonic, the nomogram should be considered hypothesis-generating rather than ready for clinical use.

Accumulating evidence indicates that SGLT2i may exert beneficial effects on DR through multiple interconnected mechanisms. Retinal microvascular pericytes and mesangial cells express SGLT2, indicating potential direct targets of SGLT2i (31). Experimental studies show that SGLT2i reduce inflammatory cytokine release, reactive oxygen species (ROS) production, and glucotoxic stress, while helping preserve insulin signaling and microvascular function (7, 32–34). They may also protect tight junction proteins, reduce vascular leakage, maintain adiponectin signaling, and inhibit VEGF-related angiogenesis and permeability (33, 35–38). In addition, SGLT2i may alleviate retinal hypoxia through mild ketosis and preserve neurovascular unit function, including reduced glial activation and retinal cell apoptosis (8, 36, 39). These experimental findings lend biological plausibility to the association observed in our cohort. However, the present study could not determine whether OCTA-derived vascular changes explain or mediate this treatment-associated reduction in DR progression.

MR analysis indicated that genetically proxied variation at the SLC5A2 locus was associated with an increased risk of DR. This supports possible involvement of the SGLT2 pathway, but the direction of the MR estimate should be interpreted cautiously and should not be regarded as directly equivalent to the pharmacologic effect of SGLT2 inhibitors. Several plasma proteins and circulating metabolites showed nominal mediation effects. However, most signals did not survive multiple-testing correction. Although some candidates are broadly consistent with previously reported effects of SGLT2i on oxidative stress, inflammation, calcium signaling, ketone-body metabolism, lipid utilization, and other aspects of systemic metabolic reprogramming (34, 40–46),these findings remain preliminary and should be interpreted as hypothesis-generating rather than definitive evidence of mechanistic pathways.

Several limitations should be acknowledged. First, the retrospective single-center design and relatively short follow-up may introduce residual confounding, selection bias, and limited long-term interpretability. The median follow-up of 17.7 months is relatively short for evaluating long-term DR progression, particularly in milder NPDR, although our cohort mainly included eyes with moderate-to-advanced DR at baseline and 28.8% of eyes showed clinically detectable progression during follow-up. Treatment was not randomized, and confounding by indication remains possible. Although IPTW based on the five multiply imputed datasets yielded consistent results and substantially improved baseline balance, a modest residual imbalance remained for DN, and residual confounding cannot be fully excluded. Second, the limited sample size and number of progression events may have reduced statistical power and increased the risk of overfitting, particularly for the multivariable Cox models and nomogram development. Although bootstrap-based internal validation was performed, the optimism-corrected discrimination was modest, calibration was not optimal, and external validation is still required before clinical application. Third, both eyes from some participants were included, which may introduce inter-eye correlation and requires conservative interpretation of the eye-level Cox analysis. However, cluster-robust modeling and repeated one-eye-per-participant analyses yielded directionally consistent results. Fourth, the MR and mediation findings were exploratory and require validation. Fifth, because all participants received insulin combination therapy, generalizability to broader T2DM populations may be limited. Nevertheless, our findings suggest that among insulin-treated patients with T2DM, SGLT2i use may be associated with lower DR progression risk, supporting further evaluation in larger prospective studies and randomized clinical trials.

## 5 Conclusion

In conclusion, SGLT2i use was associated with a lower risk of DR progression than sulfonylurea use among insulin-treated patients with T2DM, with consistent results across sensitivity analyses. OCTA-derived biomarkers may provide additional prognostic information but require validation. Given the study limitations, these findings should be interpreted cautiously and confirmed in larger prospective studies and randomized trials.

## Data Availability

The data underlying the retrospective cohort component of this study are not publicly available because they contain potentially identifiable patient information and are subject to institutional and ethical restrictions, but may be available from the corresponding author upon reasonable request and with permission from the relevant institution. The summary-level data used for the Mendelian randomization analyses were obtained from publicly available genome-wide association study datasets, as cited in the manuscript. Requests to access the datasets should be directed to 2020120208@mail.sdu.edu.cn.

## Supplementary material

Supplementary Figure 1 Representative SS-OCTA images showing vascular density, perfusion area, choroidal vascular volume, choroidal vascularity index, and retinal/choroidal thickness measurements based on the ETDRS grid. CCP, choriocapillaris plexus; CVI, choroidal vascularity index; CVV, choroidal vascular volume; DCP, deep capillary plexus; ICP, intermediate capillary plexus; SCP, superficial capillary plexus; GCC, ganglion cell complex; GCL+IPL, ganglion cell layer plus inner plexiform layer; RNFL, retinal nerve fiber layer; SS-OCTA, swept-source optical coherence tomography angiography; ETDRS, Early Treatment Diabetic Retinopathy Study.

Supplementary Figure 2 Baseline sectoral OCTA differences between eyes with and without DME. **(A)** Retinal VD in the SCP, ICP, and DCP. **(B)** Retinal and choroidal perfusion area in the SCP, ICP, DCP, and CCP. **(C)** CVV and CVI. **(D)** Retinal and choroidal thickness, including GCC, RNFL, GCL+IPL, and choroid. Sectoral significance maps are presented according to the ETDRS grid. Gray indicates P ≥ 0.05, light orange indicates P < 0.05, and dark orange indicates P < 0.001. Asterisks indicate statistically significant sectors. The color scale reflects the level of statistical significance rather than the direction of difference. Orientation markers indicate the S, I, N, and T directions. CCP, choriocapillaris plexus; CVI, choroidal vascularity index; CVV, choroidal vascular volume; DCP, deep capillary plexus; DME, diabetic macular edema; ETDRS, Early Treatment Diabetic Retinopathy Study; GCC, ganglion cell complex; GCL+IPL, ganglion cell layer plus inner plexiform layer; ICP, intermediate capillary plexus; OCTA, optical coherence tomography angiography; RNFL, retinal nerve fiber layer; SCP, superficial capillary plexus; VD, vessel density.

Supplementary Figure 3 Exploratory nomogram for predicting 12-month and 24-month survival probabilities based on Model 3. The nomogram was constructed using treatment group, sex, age, diabetes duration, severity of diabetic retinopathy, HbA1c, diabetic nephropathy, vessel density of the superficial capillary plexus, and vessel density of the deep capillary plexus. For each predictor, the corresponding score is assigned according to the points scale at the top; the total score is then used to estimate the 12-month and 24-month survival probabilities. DCP, deep capillary plexus; DN, diabetic nephropathy; DR, diabetic retinopathy; DM Dur, diabetes duration; HbA1c, glycated hemoglobin; INS, insulin; Mod NPDR, moderate non-proliferative diabetic retinopathy; PDR, proliferative diabetic retinopathy; SCP, superficial capillary plexus; Sev NPDR, severe non-proliferative diabetic retinopathy; SGLT2i, sodium-glucose cotransporter 2 inhibitor; SUL, sulfonylurea; VD, vessel density.

Supplementary Figure 4 Calibration plots of the exploratory prediction model for 12-month and 24-month survival probabilities of DR progression. **(A)** Calibration plot for 12-month progression-free survival probability. **(B)** Calibration plot for 24-month progression-free survival probability. The dashed diagonal line represents perfect agreement between predicted and observed probabilities. Points indicate grouped observed probabilities, with error bars showing 95% confidence intervals. The rug plots above the x-axis show the distribution of predicted probabilities. Abbreviations: DR, diabetic retinopathy.

Supplementary Figure 5 Exploratory decision curve analysis at the 24-month follow-up for model comparison. **(A)** Net benefit of the treat-none strategy (light gray curve), treat-all strategy (dark gray curve), Model 1 (blue curve), and Model 3 (red curve). Model 3 showed higher net benefit than Model 1 across threshold probabilities below 0.40 and also outperformed the treat-all strategy within this range. **(B)** Net benefit of the treat-none strategy (light gray curve), treat-all strategy (dark gray curve), Model 2 (green curve), and Model 3 (red curve). Model 3 showed higher net benefit than Model 2 at threshold probabilities above 0.25 and maintained an advantage over the treat-all strategy up to a threshold probability of 0.40. Decision curves were derived from Cox regression models.
