# Supplementary figures and images for: SGLT2 inhibitors and diabetic retinopathy progression: evidence from a retrospective cohort study and Mendelian randomization analysis
Source: Front Endocrinol (Lausanne). 2026 Jul 16;17:1846454. doi: 10.3389/fendo.2026.1846454 (PMC13422178; doi:10.3389/fendo.2026.1846454)

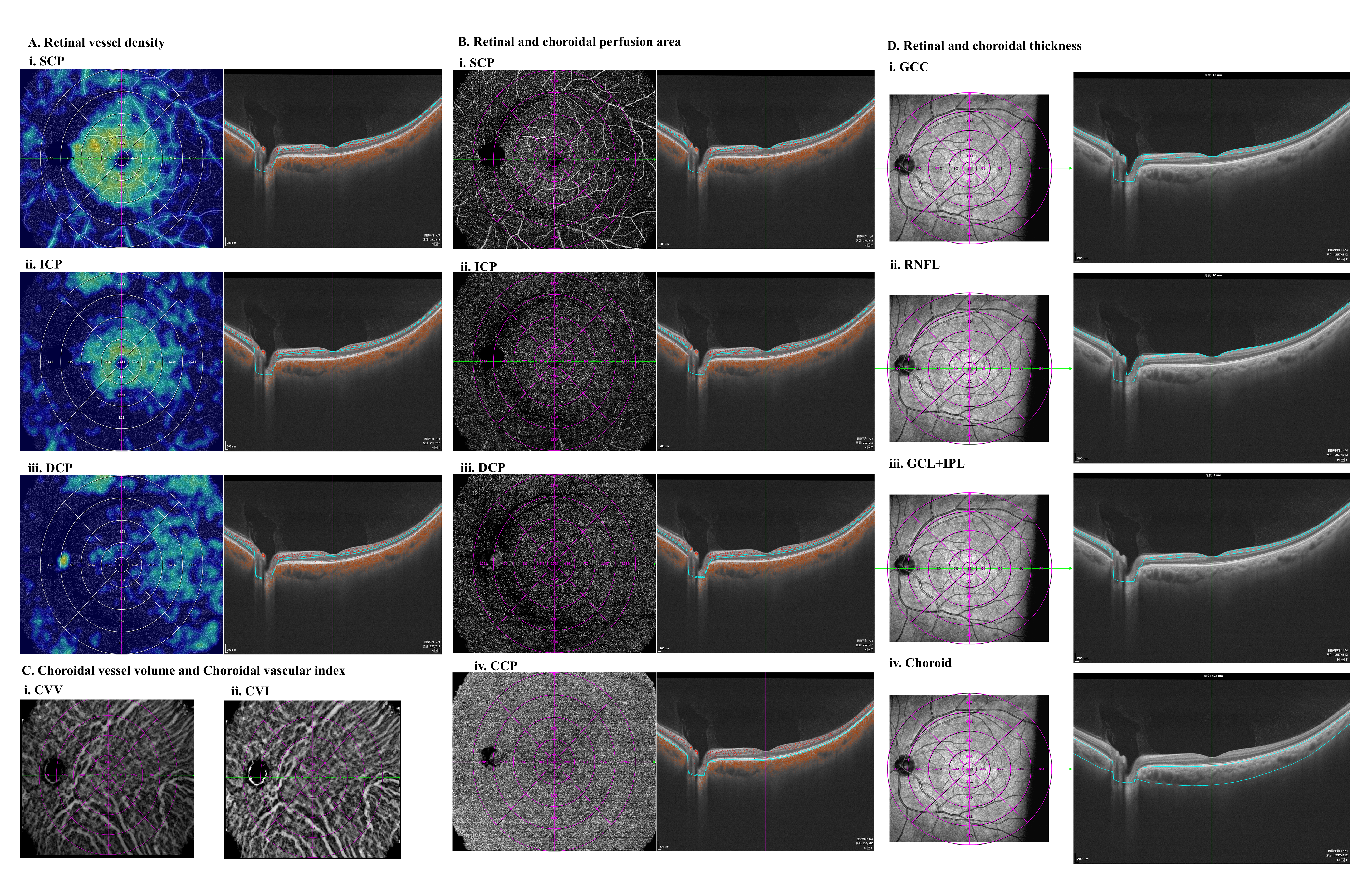

Supplement: Supplementary Figure 1 — Representative SS-OCTA images showing vascular density, perfusion area, choroidal vascular volume, choroidal vascularity index, and retinal/choroidal thickness measurements based on the ETDRS grid. CCP, choriocapillaris plexus; CVI, choroidal vascularity index; CVV, choroidal vascular volume; DCP, deep capillary plexus; ICP, intermediate capillary plexus; SCP, superficial capillary plexus; GCC, ganglion cell complex; GCL+IPL, ganglion cell layer plus inner plexiform layer; RNFL, retinal nerve fiber layer; SS-OCTA, swept-source optical coherence tomography angiography; ETDRS, Early Treatment Diabetic Retinopathy Study. [file Image1.tif]

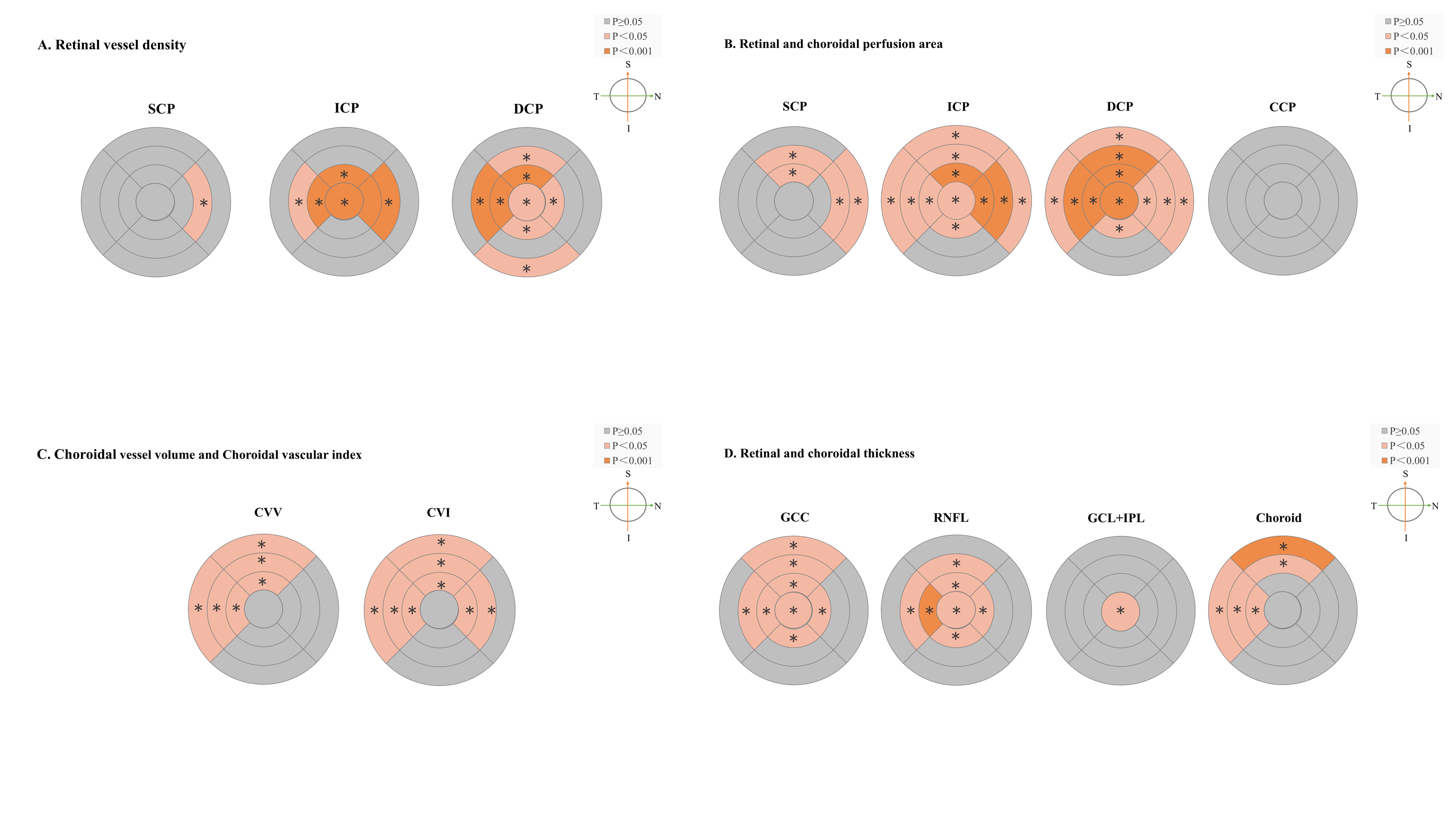

Supplement: Supplementary Figure 2 — Baseline sectoral OCTA differences between eyes with and without DME. (A) Retinal VD in the SCP, ICP, and DCP. (B) Retinal and choroidal perfusion area in the SCP, ICP, DCP, and CCP. (C) CVV and CVI. (D) Retinal and choroidal thickness, including GCC, RNFL, GCL+IPL, and choroid. Sectoral significance maps are presented according to the ETDRS grid. Gray indicates P ≥ 0.05, light orange indicates P < 0.05, and dark orange indicates P < 0.001. Asterisks indicate statistically significant sectors. The color scale reflects the level of statistical significance rather than the direction of difference. Orientation markers indicate the S, I, N, and T directions. CCP, choriocapillaris plexus; CVI, choroidal vascularity index; CVV, choroidal vascular volume; DCP, deep capillary plexus; DME, diabetic macular edema; ETDRS, Early Treatment Diabetic Retinopathy Study; GCC, ganglion cell complex; GCL+IPL, ganglion cell layer plus inner plexiform layer; ICP, intermediate capillary plexus; OCTA, optical coherence tomography angiography; RNFL, retinal nerve fiber layer; SCP, superficial capillary plexus; VD, vessel density. [file Image2.tif]

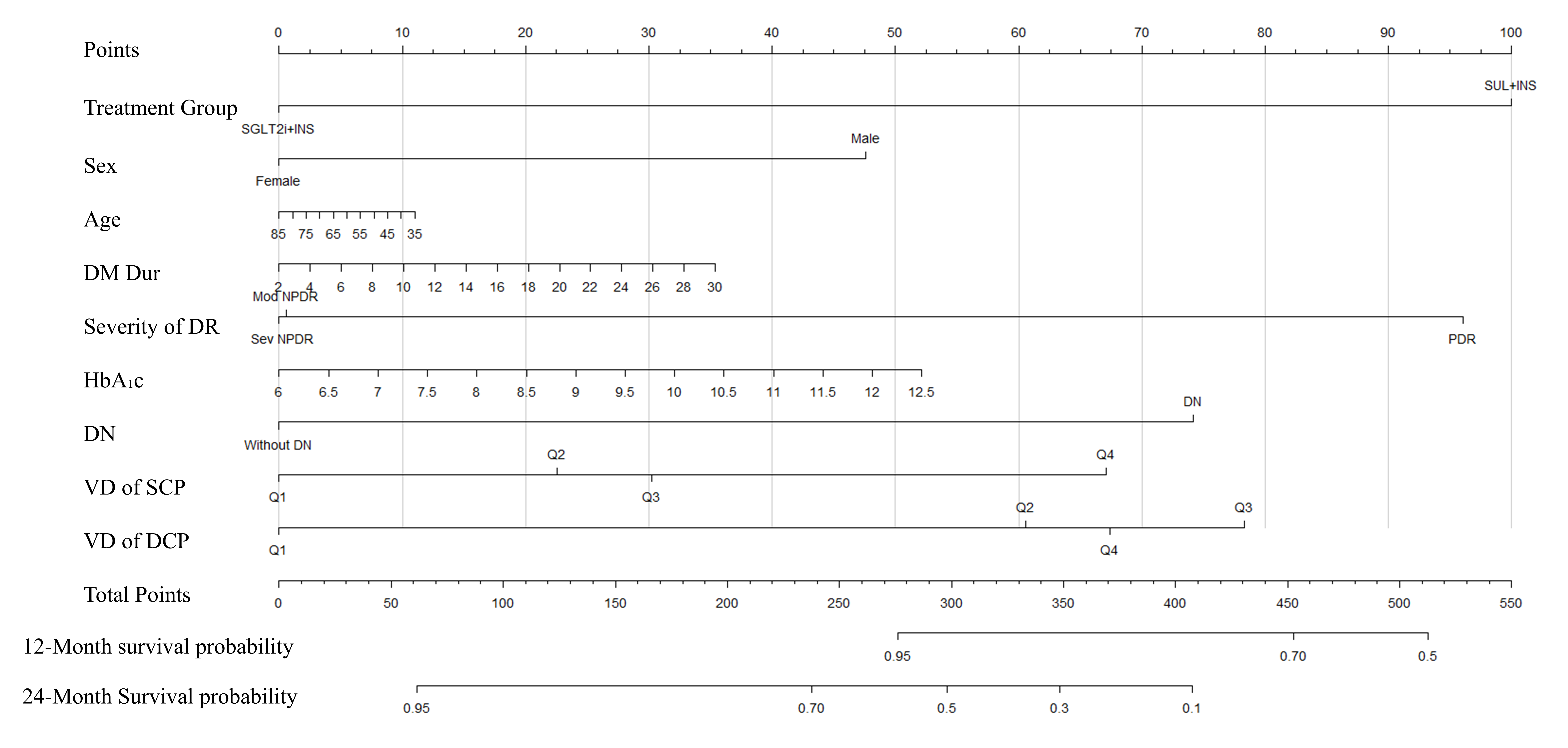

Supplement: Supplementary Figure 3 — Exploratory nomogram for predicting 12-month and 24-month survival probabilities based on Model 3. The nomogram was constructed using treatment group, sex, age, diabetes duration, severity of diabetic retinopathy, HbA1c, diabetic nephropathy, vessel density of the superficial capillary plexus, and vessel density of the deep capillary plexus. For each predictor, the corresponding score is assigned according to the points scale at the top; the total score is then used to estimate the 12-month and 24-month survival probabilities. DCP, deep capillary plexus; DN, diabetic nephropathy; DR, diabetic retinopathy; DM Dur, diabetes duration; HbA1c, glycated hemoglobin; INS, insulin; Mod NPDR, moderate non-proliferative diabetic retinopathy; PDR, proliferative diabetic retinopathy; SCP, superficial capillary plexus; Sev NPDR, severe non-proliferative diabetic retinopathy; SGLT2i, sodium-glucose cotransporter 2 inhibitor; SUL, sulfonylurea; VD, vessel density. [file Image3.tif]

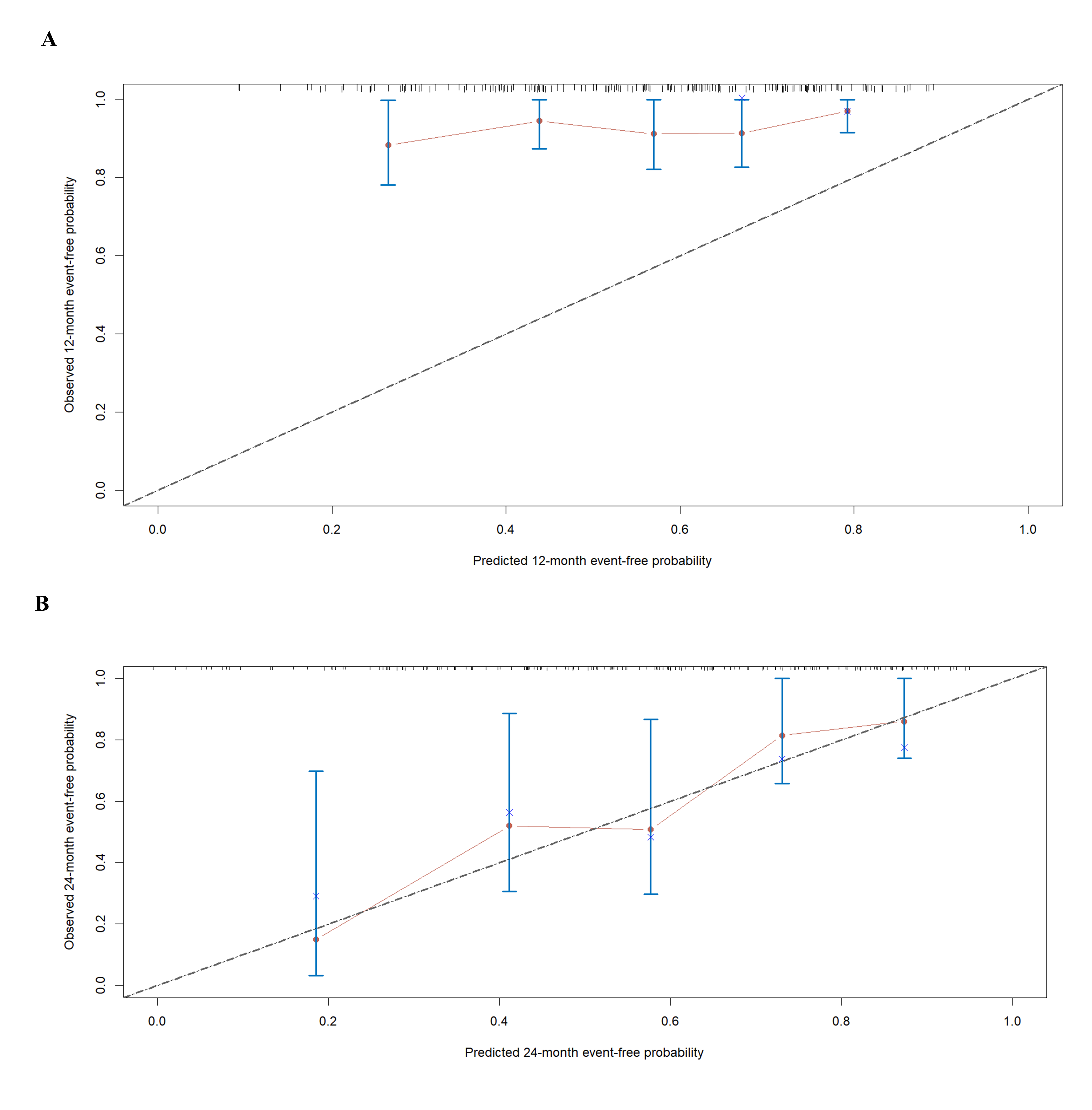

Supplement: Supplementary Figure 4 — Calibration plots of the exploratory prediction model for 12-month and 24-month survival probabilities of DR progression. (A) Calibration plot for 12-month progression-free survival probability. (B) Calibration plot for 24-month progression-free survival probability. The dashed diagonal line represents perfect agreement between predicted and observed probabilities. Points indicate grouped observed probabilities, with error bars showing 95% confidence intervals. The rug plots above the x-axis show the distribution of predicted probabilities. Abbreviations: DR, diabetic retinopathy. [file Image4.tif]

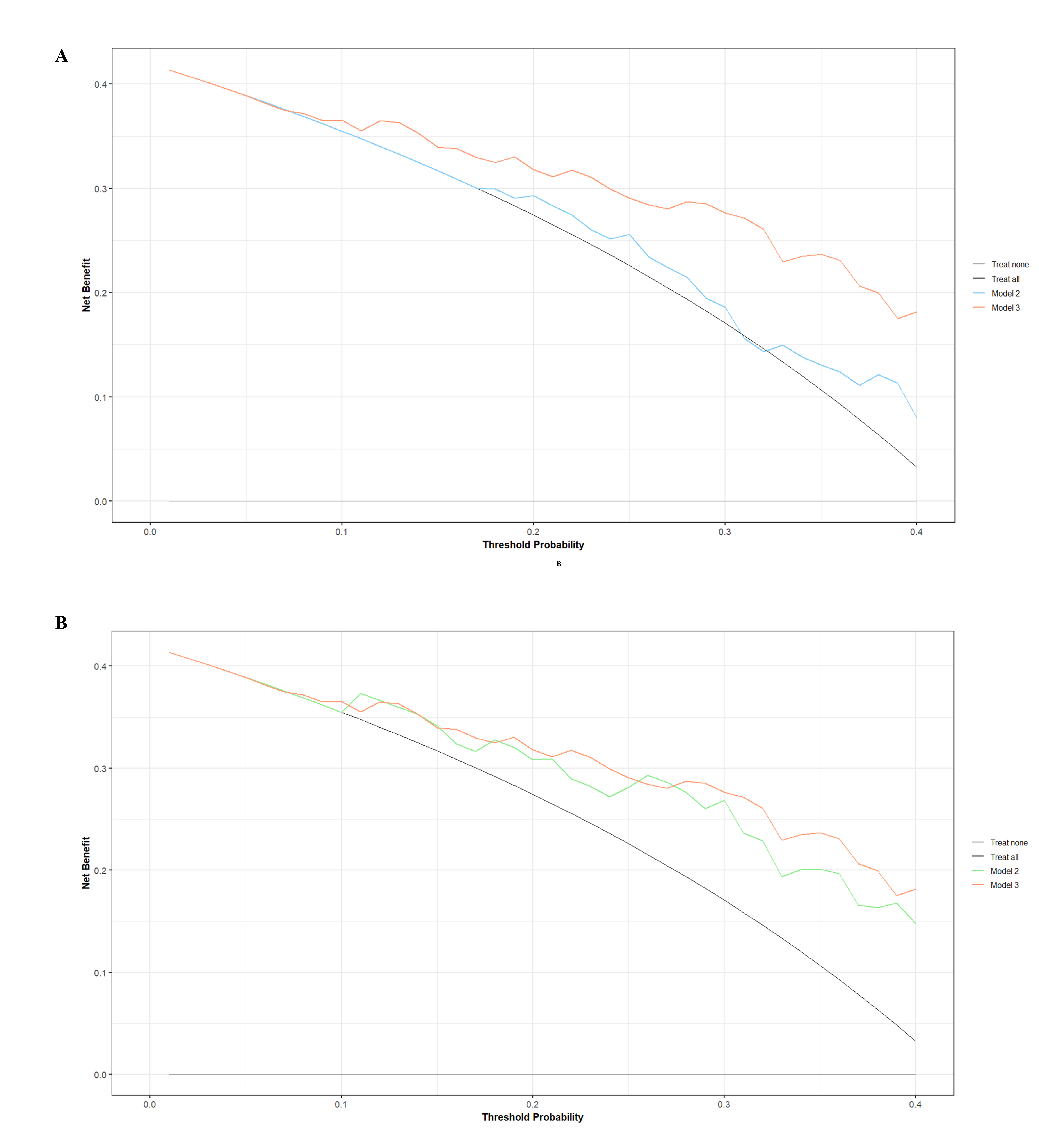

Supplement: Supplementary Figure 5 — Exploratory decision curve analysis at the 24-month follow-up for model comparison. (A) Net benefit of the treat-none strategy (light gray curve), treat-all strategy (dark gray curve), Model 1 (blue curve), and Model 3 (red curve). Model 3 showed higher net benefit than Model 1 across threshold probabilities below 0.40 and also outperformed the treat-all strategy within this range. (B) Net benefit of the treat-none strategy (light gray curve), treat-all strategy (dark gray curve), Model 2 (green curve), and Model 3 (red curve). Model 3 showed higher net benefit than Model 2 at threshold probabilities above 0.25 and maintained an advantage over the treat-all strategy up to a threshold probability of 0.40. Decision curves were derived from Cox regression models. [file Image5.tif]
