# Supplementary material for: SGLT2 inhibitors and diabetic retinopathy progression: evidence from a retrospective cohort study and Mendelian randomization analysis
Source: Front Endocrinol (Lausanne). 2026 Jul 16;17:1846454. doi: 10.3389/fendo.2026.1846454 (PMC13422178; doi:10.3389/fendo.2026.1846454)
Supplement: Supplementary file 6 [file Table1.docx]

Supplementary Tables

[Supplementary Table S1. Retinal and choroidal segmentation boundaries used for OCTA analysis. 2](#_Toc232530978)

[Supplementary Table S2. Characteristics of the summary datasets for the SGLT2 target, candidate mediators, and DR used in the Mendelian randomization analyses. 3](#_Toc232530979)

[Supplementary Table S3. Baseline characteristics of included and excluded patients. 4](#_Toc232530980)

[Supplementary Table S4. Comparison of retinal vessel density between eyes with and without DME. 6](#_Toc232530981)

[Supplementary Table S5. Comparison of retinal and choroidal perfusion area between eyes with and without DME. 8](#_Toc232530982)

[Supplementary Table S6. Comparison of CVV and CVI between eyes with and without DME. 11](#_Toc232530983)

[Supplementary Table S7. Comparison of retinal and choroidal thickness between eyes with and without DME. 13](#_Toc232530984)

[Supplementary Table S8. Univariate Cox regression analyses of factors associated with DR progression and DME development. 16](#_Toc232530985)

[Supplementary Table S9. Collinearity diagnosis of risk factors for diabetic retinopathy progression. 18](#_Toc232530986)

[Supplementary Table S10. Tests of proportional hazards assumption for Model 3. 19](#_Toc232530987)

[Supplementary Table S11. Descriptive statistics for DN and HbA1c before and after multiple imputation. 20](#_Toc232530988)

[Supplementary Table S12. Covariate balance before and after inverse probability of treatment weighting. 21](#_Toc232530989)

[Supplementary Table S13. Comparison of candidate models and bootstrap-based internal validation of the exploratory prediction model for diabetic retinopathy progression. 23](#_Toc232530990)

[Supplementary Table S14. Mendelian randomization analysis of the causal association between the SGLT2 target (SLC5A2) and DR. 24](#_Toc232530991)

[Supplementary Table S15. Heterogeneity and horizontal pleiotropy in Mendelian randomization analyses of the SGLT2 target (SLC5A2) and DR. 25](#_Toc232530992)

[Supplementary Table S16. Mediating effects of the SGLT2 target (SLC5A2) on diabetic retinopathy via plasma proteins and circulating metabolites. 26](#_Toc232530993)

[Supplementary Table S17. Heterogeneity and horizontal pleiotropy in two-step Mendelian randomization mediation analyses of the SGLT2 target (SLC5A2) and DR. 28](#_Toc232530994)

# Supplementary Table S1. Retinal and choroidal segmentation boundaries used for OCTA analysis.

| Structure | Upper Boundary | Lower Boundary |
| --- | --- | --- |
| RNFL | ILM - 5 | NFL/GCL |
| GCL+IPL | NFL/GCL | IPL/INL - max ((IPL/INL - NFL/GCL)/3, 10) |
| GCC | ILM - 5 | IPL/INL – max ((IPL/INL - NFL/GCL)/3, 10） |
| SCP | NFL/GCL | IPL/INL – max ((IPL/INL - NFL/GCL)/3, 10) |
| ICP | IPL/INL – max ((IPL/INL - NFL/GCL)/3, 10) | IPL/INL + (INL/OPL - IPL/INL)/2 |
| DCP | IPL/INL + (INL/OPL - IPL/INL)/2 | INL/OPL + 25 |
| CCP | BM - 10 | BM + 25 |
| Choroid | BM + 25 | Choroid lower boundary |

**Note:** Segmentation boundaries were based on the built-in segmentation settings of the SS-OCTA system (VG100D, SVision Imaging, Ltd.; version 3.1.255). All offsets are expressed in μm.

**Abbreviations:** BM, Bruch’s membrane; CCP, choriocapillaris plexus; DCP, deep capillary plexus; GCC, ganglion cell complex; GCL+IPL, ganglion cell layer plus inner plexiform layer; ICP, intermediate capillary plexus; ILM, internal limiting membrane; INL/OPL, boundary between the inner nuclear layer and outer plexiform layer; IPL/INL, boundary between the inner plexiform layer and inner nuclear layer; NFL/GCL, boundary between the retinal nerve fiber layer and ganglion cell layer; OCTA, optical coherence tomography angiography; RNFL, retinal nerve fiber layer; SCP, superficial capillary plexus; SS-OCTA, swept-source optical coherence tomography angiography.

# Supplementary Table S2. Characteristics of the summary datasets for the SGLT2 target, candidate mediators, and DR used in the Mendelian randomization analyses.

| Trait | | Resource | Dataset | Sample size | Population | PMID | DOI/URL |
| --- | --- | --- | --- | --- | --- | --- | --- |
| SGLT2 | | IEU OpenGWAS | eqtl-a-ENSG00000140675 | 31,306 | European | NA | https://opengwas.io/datasets/eqtl-a-ENSG00000140675 |
| DR | | Finn R12 | finngen_R12_DM_RETINOPATHY | 13,167 cases/ 131,272controls | European | NA | https://storage.googleapis.com/finngen- public-data-r12/summary_stats/release/finngen_R12_DM_RETINOPATHY.gz |
| Plasma proteins | deCODE Genetics | Plasma pQTL Study | 35,559 | European | 34857953 | NA |  |
| Circulating metabolites | | IEU OpenGWAS | GCST90199621 - GCST9020120 | 121,000 | European | 36635386 | NA |

**Abbreviations:** DR, diabetic retinopathy; eQTL, expression quantitative trait locus; GWAS, genome-wide association study; IEU, MRC Integrative Epidemiology Unit; MR, Mendelian randomization; NMR, nuclear magnetic resonance; pQTL, protein quantitative trait locus; SGLT2, sodium-glucose cotransporter 2.

# Supplementary Table S3. Baseline characteristics of included and excluded patients.

| Characteristics | Included (N = 191) | |  | Excluded (N = 29) | | P value |
| --- | --- | --- | --- | --- | --- | --- |
|  | N | Mean (SD) or % |  | N | Mean (SD) or % |  |
| Age (year) | 191 | 58.99 (1.04) |  | 29 | 60.31 (2.17) | 0.583 |
| Sex | 191 |  |  | 29 |  | 0.909 |
| Female | 62 | 32.5 |  | 8 | 27.6 |  |
| Male | 129 | 67.5 |  | 21 | 72.4 |  |
| Duration of diabetes (year) | 191 | 13.96 (0.78) |  | 29 | 14.66 (1.54) | 0.687 |
| Body mass index (kg/m²) | 141 | 25.49 (0.34) |  | 18 | 22.79 (0.87) | **0.004** |
| logMAR | 184 | 0.41 (0.03) |  | 29 | 0.38 (0.09) | 0.713 |
| Severity of DR |  |  |  |  |  |  |
| Mild NPDR | 0 | 0 |  | 3 | 10.3 | **0.015** |
| Moderate NPDR | 59 | 30.9 |  | 18 | 62.1 |  |
| Severe NPDR | 112 | 58.6 |  | 2 | 6.9 |  |
| PDR | 20 | 10.5 |  | 6 | 20.7 |  |
| HbA1c (%) | 180 | 8.81 (0.16) |  | 23 | 8.20 (0.53) | 0.272 |
| Self-reported comorbidities (yes） |  |  |  |  |  |  |
| Hypertension | 104 | 60.5 |  | 14 | 48.3 | 0.542 |
| Hyperlipidemia | 97 | 52.2 |  | 17 | 58.6 | 0.793 |
| DN | 84 | 46.2 |  | 8 | 28.6 | 0.150 |
| Smoke |  |  |  |  |  |  |
| Never | 115 | 69.2 |  | 15 | 83.3 | 0.587 |
| Former | 21 | 12.4 |  | 1 | 5.8 |  |
| Current | 31 | 18.3 |  | 2 | 11.1 |  |
| SS-OCTA Metrics ^a^ |  |  |  |  |  |  |
| Superficial Capillary Plexus |  |  |  |  |  |  |
| Vessel density (%) | 191 | 44.27 (0.61) |  | 27 | 44.73 (1.28) | 0.747 |
| Perfusion area (mm²) | 191 | 11.18 (0.16) |  | 27 | 11.31 (0.40) | 0.762 |
| Deep Capillary Plexus |  |  |  |  |  |  |
| Vessel density (%) | 191 | 5.00 (0.38) |  | 27 | 5.66 (1.11) | 0.577 |
| Perfusion area (mm²) | 191 | 5.02 (0.14) |  | 27 | 5.14 (0.35) | 0.768 |
| Macular retinal thickness  (6 × 6) (μm) | 191 | 318.25 (3.32) |  | 27 | 332.39 (13.81) | 0.320 |

**Note:** ^a^ SS-OCTA metrics were derived only from the macular-centered 6 × 6mm field to facilitate inter-group quality control.

**Abbreviations:**DR, diabetic retinopathy; DN, diabetic nephropathy; HbA1c, glycated hemoglobin; logMAR, logarithm of the minimum angle of resolution; NPDR, non-proliferative diabetic retinopathy; PDR, proliferative diabetic retinopathy; SD, standard deviation; SS-OCTA, swept-source optical coherence tomography angiography.

# Supplementary Table S4. Comparison of retinal vessel density between eyes with and without DME.

| Region |  | Eyes without DME  (N = 147) | | Eyes with DME  (N = 44) | | P value |
| --- | --- | --- | --- | --- | --- | --- |
|  |  | Mean (SD) | | Mean (SD) | |  |
| **SCP vessel density (%)** | |  | |  | |  |
| Total |  |  |  |  |  |  |
|  | Circle 0-3 mm |  | 44.34 (0.87) |  | 44.94 (2.23) | 0.803 |
|  | Circle 0-6 mm |  | 44.39 (0.67) |  | 44.02 (1.32) | 0.804 |
|  | Circle 0-9 mm |  | 32.97 (0.49) |  | 32.61 (0.83) | 0.706 |
|  | Circle 0-12 mm |  | 25.76 (0.36) |  | 25.98 (0.64) | 0.764 |
|  | Ring 3-6 mm |  | 44.41 (0.66) |  | 43.73 (1.18) | 0.617 |
|  | Ring 6-9 mm |  | 23.84 (0.45) |  | 23.42 (0.82) | 0.645 |
|  | Ring 9-12 mm |  | 16.44 (0.40) |  | 17.16 (0.82) | 0.43 |
| Superior |  |  |  |  |  |  |
|  | Ring 3-6 mm |  | 46.05 (0.82) |  | 42.41 (1.66) | 0.500 |
|  | Ring 6-9 mm |  | 29.88 (0.61) |  | 27.07 (1.46) | 0.075 |
|  | Ring 9-12 mm |  | 21.26 (0.57) |  | 21.28 (1.24) | 0.989 |
| Temporal |  |  |  |  |  |  |
|  | Ring 3-6 mm |  | 40.53 (0.84) |  | 42.50 (1.93) | 0.351 |
|  | Ring 6-9 mm |  | 24.13 (0.68) |  | 25.61 (1.29) | 0.311 |
|  | Ring 9-12 mm |  | 14.49 (0.51) |  | 15.25 (1.40) | 0.611 |
| Inferior |  |  |  |  |  |  |
|  | Ring 3-6 mm |  | 42.17 (0.87) |  | 42.97 (1.41) | 0.278 |
|  | Ring 6-9 mm |  | 22.97 (0.71) |  | 25.39 (1.43) | 0.129 |
|  | Ring 9-12 mm |  | 18.80 (0.70) |  | 21.89 (1.64) | 0.084 |
| Nasal |  |  |  |  |  |  |
|  | Ring 3-6 mm |  | 48.79 (0.92) |  | 45.70 (1.52) | 0.084 |
|  | Ring 6-9 mm |  | 18.39 (0.76) |  | 15.28 (1.31) | **0.039** |
|  | Ring 9-12 mm |  | 11.25 (0.58) |  | 10.33 (1.01) | 0.428 |
| **ICP vessel density (%)** | |  |  |  |  |  |
| Total |  |  |  |  |  |  |
|  | Circle 0-3 mm |  | 26.45 (0.92) |  | 19.66 (1.71) | **<0.001** |
|  | Circle 0-6 mm |  | 22.13 (0.79) |  | 15.84 (1.41) | **<0.001** |
|  | Circle 0-9 mm |  | 16.38 (0.56) |  | 12.40 (1.07) | **0.001** |
|  | Circle 0-12 mm |  | 13.92 (0.42) |  | 11.38 (0.89) | **0.010** |
|  | Ring 3-6 mm |  | 14.59 (0.79) |  | 14.58 (1.43) | **<0.001** |
|  | Ring 6-9 mm |  | 11.79 (0.45) |  | 9.62 (0.99) | **0.045** |
|  | Ring 9-12 mm |  | 10.73 (0.43) |  | 1.00 (0.90) | 0.464 |
| Superior |  |  |  |  |  |  |
|  | Ring 3-6 mm |  | 18.85 (0.93) |  | 10.82 (1.53) | **<0.001** |
|  | Ring 6-9 mm |  | 12.61 (0.67) |  | 9.28 (1.58) | 0.053 |
|  | Ring 9-12 mm |  | 12.14 (0.61) |  | 11.51 (1.52) | 0.700 |
| Temporal |  |  |  |  |  |  |
|  | Ring 3-6 mm |  | 29.62 (0.93) |  | 22.62 (1.96) | **0.001** |
|  | Ring 6-9 mm |  | 21.84 (0.89) |  | 17.19 (1.67) | **0.014** |
|  | Ring 9-12 mm |  | 15.13 (0.82) |  | 13.20 (1.58) | 0.278 |
| Inferior |  |  |  |  |  |  |
|  | Ring 3-6 mm |  | 16.85 (0.92) |  | 13.67 (1.78) | 0.112 |
|  | Ring 6-9 mm |  | 9.21 (0.53) |  | 9.43 (1.01) | 0.843 |
|  | Ring 9-12 mm |  | 11.56 (0.74) |  | 11.97 (1.36) | 0.792 |
| Nasal |  |  |  |  |  |  |
|  | Ring 3-6 mm |  | 17.65 (0.93) |  | 10.92 (1.31) | **<0.001** |
|  | Ring 6-9 mm |  | 3.58 (0.32) |  | 1.98 (0.36) | **0.001** |
|  | Ring 9-12 mm |  | 4.15 (0.37) |  | 2.93 (0.52) | 0.057 |
|  |  |  |  |  |  |  |
| **DCP vessel density (%)** | |  |  |  |  |  |
| Total |  |  |  |  |  |  |
|  | Circle 0-3 mm |  | 4.67 (0.51) |  | 2.40 (0.56) | **0.003** |
|  | Circle 0-6 mm |  | 5.51 (0.43) |  | 2.84 (0.45) | **<0.001** |
|  | Circle 0-9 mm |  | 6.05 (0.37) |  | 3.82 (0.45) | **<0.001** |
|  | Circle 0-12 mm |  | 5.83 (0.34) |  | 3.92 (0.40) | **<0.001** |
|  | Ring 3-6 mm |  | 5.92 (0.46) |  | 2.96 (0.48) | **<0.001** |
|  | Ring 6-9 mm |  | 6.49 (0.39) |  | 4.59 (0.54) | **0.005** |
|  | Ring 9-12 mm |  | 5.50 (0.36) |  | 4.03 (0.49) | **0.016** |
| Superior |  |  |  |  |  |  |
|  | Ring 3-6 mm |  | 4.35 (0.45) |  | 1.45 (0.35) | **<0.001** |
|  | Ring 6-9 mm |  | 4.24 (0.46) |  | 2.47 (0.72) | **0.037** |
|  | Ring 9-12 mm |  | 4.17 (0.40) |  | 3.15 (0.54) | 0.129 |
| Temporal |  |  |  |  |  |  |
|  | Ring 3-6 mm |  | 12.24 (0.92) |  | 6.72 (1.17) | **<0.001** |
|  | Ring 6-9 mm |  | 13.25 (0.99) |  | 7.77 (1.37) | **0.001** |
|  | Ring 9-12 mm |  | 10.67 (0.90) |  | 7.99 (1.38) | 0.105 |
| Inferior |  |  |  |  |  |  |
|  | Ring 3-6 mm |  | 3.97 (0.49) |  | 2.34 (0.61) | **0.037** |
|  | Ring 6-9 mm |  | 2.59 (0.31) |  | 1.69 (0.36) | 0.063 |
|  | Ring 9-12 mm |  | 3.58 (0.47) |  | 2.19 (0.47) | **0.036** |
| Nasal |  |  |  |  |  |  |
|  | Ring 3-6 mm |  | 2.58 (0.29) |  | 1.37 (0.35) | **0.008** |
|  | Ring 6-9 mm |  | 5.58 (0.34) |  | 6.20 (0.73) | 0.445 |
|  | Ring 9-12 mm |  | 3.25 (0.25) |  | 2.57 (0.45) | 0.192 |

**Note:**Data are presented as mean (SD). Analyses were performed at the eye level.
**Abbreviations:**DCP, deep capillary plexus; DME, diabetic macular edema; ICP, intermediate capillary plexus; SCP, superficial capillary plexus; SD, standard deviation.

| Supplementary Table S5. Comparison of retinal and choroidal perfusion area between eyes with and without DME. | | | | | | |
| --- | --- | --- | --- | --- | --- | --- |
| Region |  | Eyes without DME  (N = 147) | | Eyes with DME  (N = 44) | | P value |
|  |  | Mean (SD) | | Mean (SD) | | |
| **SCP Perfusion Area (mm²)** | |  |  |  |  |  |
| Total |  |  |  |  |  |  |
|  | Circle 0-3 mm |  | 2.70 (0.05) | 2.68 (0.15) | | 0.870 |
|  | Circle 0-6 mm |  | 11.24 (0.17) | 10.98 (0.36) | | 0.506 |
|  | Circle 0-9 mm |  | 21.18 (0.28) | 20.49 (0.54) | | 0.264 |
|  | Circle 0-12 mm | | 32.63 (0.41) | 31.41 (0.80) | | 0.175 |
|  | Circle 3-6 mm |  | 8.54 (0.13) | 8.30 (0.23) | | 0.367 |
|  | Circle 6-9 mm |  | 9.93 (0.13) | 9.51 (0.26) | | 0.140 |
|  | Circle 9-12 mm | | 11.45 (0.16) | 10.91 (0.38) | | 0.195 |
| Superior |  |  |  |  |  |  |
|  | Circle 3-6 mm |  | 2.21 (0.04) | 2.04 (0.08) | | **0.044** |
|  | Circle 6-9 mm |  | 2.85 (0.04) | 2.62 (0.10) | | **0.036** |
|  | Circle 9-12 mm | | 3.38 (0.05) | 3.24 (0.09) | | 0.162 |
| Temporal |  |  |  |  |  |  |
|  | Circle 3-6 mm |  | 1.90 (0.04) | 1.94 (0.08) | | 0.681 |
|  | Circle 6-9 mm |  | 2.33 (0.05) | 2.31 (0.09) | | 0.812 |
|  | Circle 9-12 mm | | 2.56 (0.05) | 2.38 (0.15) | | 0.237 |
| Inferior |  |  |  |  |  |  |
|  | Circle 3-6 mm |  | 2.07 (0.04) | 2.09 (0.06) | | 0.727 |
|  | Circle 6-9 mm |  | 2.49 (0.05) | 2.55 (0.10) | | 0.612 |
|  | Circle 9-12 mm | | 2.94 (0.08) | 2.93 (0.20) | | 0.960 |
| Nasal |  |  |  |  |  |  |
|  | Circle 3-6 mm |  | 2.35 (0.04) | 2.21 (0.06) | | 0.075 |
|  | Circle 6-9 mm |  | 2.26 (0.05) | 2.01 (0.10) | | **0.020** |
|  | Circle 9-12 mm | | 2.57 (0.05) | 2.35 (0.09) | | **0.042** |
| **ICP Perfusion Area (mm²)** | |  |  |  |  |  |
| Total |  |  |  |  |  |  |
|  | Circle 0-3 mm |  | 1.84 (0.04) | 1.55 (0.08) | | **0.001** |
|  | Circle 0-6 mm |  | 7.25 (0.16) | 6.12 (0.28) | | **<0.001** |
|  | Circle 0-9 mm |  | 14.14 (0.31) | 12.00 (0.54) | | **0.001** |
|  | Circle 0-12 mm | | 22.78 (0.50) | 19.41 (0.84) | | **0.001** |
|  | Ring 3-6 mm |  | 5.41 (0.12) | 4.57 (0.22) | | **0.001** |
|  | Ring 6-9 mm |  | 6.89 (0.16) | 5.87 (0.27) | | **0.001** |
|  | Ring 9-12 mm |  | 8.64 (0.22) | 7.41 (0.37) | | **0.004** |
| Superior |  |  |  |  |  |  |
|  | Ring 3-6 mm |  | 1.34 (0.03) | 1.07 (0.06) | | **<0.001** |
|  | Ring 6-9 mm |  | 1.90 (0.05) | 1.58 (0.09) | | **0.002** |
|  | Ring 9-12 mm |  | 2.46 (0.06) | 2.15 (0.10) | | **0.007** |
| Temporal |  |  |  |  |  |  |
|  | Ring 3-6 mm |  | 1.45 (0.03) | 1.25 (0.07) | | **0.011** |
|  | Ring 6-9 mm |  | 2.01 (0.05) | 1.69 (0.11) | | **0.006** |
|  | Ring 9-12 mm |  | 2.34 (0.06) | 1.94 (0.16) | | **0.020** |
| Inferior |  |  |  |  |  |  |
|  | Ring 3-6 mm |  | 1.26 (0.03) | 1.11 (0.06) | | **0.035** |
|  | Ring 6-9 mm |  | 1.63 (0.05) | 1.56 (0.08) | | 0.486 |
|  | Ring 9-12 mm |  | 2.13 (0.08) | 1.93 (0.16) | | 0.280 |
| Nasal |  |  |  |  |  |  |
|  | Ring 3-6 mm |  | 1.36 (0.04) | 1.13 (0.05) | | **<0.001** |
|  | Ring 6-9 mm |  | 1.34 (0.06) | 1.03 (0.06) | | **<0.001** |
|  | Ring 9-12 mm |  | 1.71 (0.07) | 1.39 (0.09) | | **0.004** |
| **DCP Perfusion Area (mm²)** | |  |  |  |  |  |
| Total |  |  |  |  |  |  |
|  | Circle 0-3 mm |  | 1.28 (0.04) | 0.98 (0.07) | | **<0.001** |
|  | Circle 0-6 mm |  | 5.27 (0.14) | 4.02 (0.26) | | **<0.001** |
|  | Circle 0-9 mm |  | 10.61 (0.29) | 8.23 (0.49) | | **<0.001** |
|  | Circle 0-12 mm | | 17.05 (0.48) | 13.34 (0.78) | | **<0.001** |
|  | Ring 3-6 mm |  | 4.00 (0.11) | 3.04 (0.20) | | **<0.001** |
|  | Ring 6-9 mm |  | 5.34 (0.16) | 4.21 (0.25) | | **<0.001** |
|  | Ring 9-12 mm |  | 6.45 (0.21) | 5.10 (0.36) | | **0.001** |
| Superior |  |  |  |  |  |  |
|  | Ring 3-6 mm |  | 1.00 (0.03) | 0.70 (0.05) | | **<0.001** |
|  | Ring 6-9 mm |  | 1.45 (0.05) | 1.10 (0.09) | | **<0.001** |
|  | Ring 9-12 mm |  | 1.81 (0.06) | 1.46 (0.10) | | **0.003** |
| Temporal |  |  |  |  |  |  |
|  | Ring 3-6 mm |  | 1.15 (0.04) | 0.87 (0.06) | | **<0.001** |
|  | Ring 6-9 mm |  | 1.70 (0.06) | 1.23 (0.12) | | **<0.001** |
|  | Ring 9-12 mm |  | 2.04 (0.09) | 1.55 (0.17) | | **0.009** |
| Inferior |  |  |  |  |  |  |
|  | Ring 3-6 mm |  | 0.91 (0.03) | 0.72 (0.06) | | **0.004** |
|  | Ring 6-9 mm |  | 1.07 (0.05) | 0.95 (0.07) | | 0.129 |
|  | Ring 9-12 mm |  | 1.37 (0.07) | 1.12 (0.12) | | 0.064 |
| Nasal |  |  |  |  |  |  |
|  | Ring 3-6 mm |  | 0.95 (0.03) | 0.75 (0.06) | | **0.001** |
|  | Ring 6-9 mm |  | 1.11 (0.04) | 0.91 (0.07) | | **0.018** |
|  | Ring 9-12 mm |  | 1.22 (0.04) | 0.95 (0.07) | | **0.001** |
| **CCP Perfusion Area (mm²)** | |  |  |  |  |  |
| Total |  |  |  |  |  |  |
|  | Circle 0-3 mm |  | 4.73 (0.08) | 4.91 (0.16) | | 0.334 |
|  | Circle 0-6 mm |  | 20.17 (0.30) | 20.58 (0.56) | | 0.515 |
|  | Circle 0-9 mm |  | 46.57 (0.60) | 47.06 (1.05) | | 0.688 |
|  | Circle 0-12 mm | | 83.81 (0.98) | 83.57 (1.83) | | 0.907 |
|  | Ring 3-6 mm |  | 15.44 (0.22) | 15.68 (0.41) | | 0.610 |
|  | Ring 6-9 mm |  | 26.39 (0.32) | 26.47 (0.52) | | 0.907 |
|  | Ring 9-12 mm |  | 37.23 (0.41) | 36.50 (0.93) | | 0.469 |
| Superior |  |  |  |  |  |  |
|  | Ring 3-6 mm |  | 3.94 (0.05) | 3.99 (0.10) | | 0.675 |
|  | Ring 6-9 mm |  | 6.76 (0.08) | 6.75 (0.21) | | 0.964 |
|  | Ring 9-12 mm |  | 9.73 (0.11) | 9.55 (0.32) | | 0.585 |
| Temporal |  |  |  |  |  |  |
|  | Ring 3-6 mm |  | 3.82 (0.06) | 3.86 (0.11) | | 0.732 |
|  | Ring 6-9 mm |  | 6.60 (0.10) | 6.61 (0.19) | | 0.975 |
|  | Ring 9-12 mm |  | 9.54 (0.12) | 9.23 (0.30) | | 0.344 |
| Inferior |  |  |  |  |  |  |
|  | Ring 3-6 mm |  | 3.91 (0.06) | 3.93 (0.12) | | 0.909 |
|  | Ring 6-9 mm |  | 6.67 (0.11) | 6.67 (0.17) | | 0.991 |
|  | Ring 9-12 mm |  | 9.11 (0.17) | 8.77 (0.50) | | 0.514 |
| Nasal |  |  |  |  |  |  |
|  | Ring 3-6 mm |  | 3.77 (0.05) | 3.91 (0.11) | | 0.270 |
|  | Ring 6-9 mm |  | 6.36 (0.08) | 6.42 (0.16) | | 0.708 |
|  | Ring 9-12 mm |  | 8.84 (0.09) | 8.92 (0.19) | | 0.692 |

**Abbreviations:**CCP, choriocapillaris plexus; DCP, deep capillary plexus; DME, diabetic macular edema; ICP, intermediate capillary plexus; SCP, superficial capillary plexus; SD, standard deviation.

# Supplementary Table S6. Comparison of CVV and CVI between eyes with and without DME.

| Region |  | Eyes without DME  (N = 147) | | Eyes with DME  (N = 44) | | P value |
| --- | --- | --- | --- | --- | --- | --- |
|  |  |  | Mean (SD) | | Mean (SD) | |
| **CVV (mm³)** | |  |  |  |  |  |
| Total |  |  |  |  |  |  |
|  | Circle 0-3 mm | | 0.64 (0.03) | | 0.56 (0.05) | 0.145 |
|  | Circle 0-6 mm | | 2.39 (0.10) | | 2.00 (0.18) | 0.063 |
|  | Circle 0-9 mm | | 4.85 (0.19) | | 3.97 (0.37) | **0.033** |
|  | Circle 0-12 mm | | 8.07 (0.29) | | 6.50 (0.60) | **0.018** |
|  | Ring 3-6 mm | | 1.74 (0.07) | | 1.44 (0.14) | **0.048** |
|  | Ring 6-9 mm | | 2.46 (0.09) | | 1.97 (0.19) | **0.019** |
|  | Ring 9-12 mm | | 3.22 (0.11) | | 2.53 (0.24) | **0.010** |
| Superior |  |  |  |  |  |  |
|  | Ring 3-6 mm | | 0.48 (0.02) | | 0.38 (0.03) | **0.013** |
|  | Ring 6-9 mm | | 0.76 (0.03) | | 0.60 (0.05) | **0.003** |
|  | Ring 9-12 mm | | 1.05 (0.03) | | 0.78 (0.07) | **0.001** |
| Temporal | |  |  |  |  |  |
|  | Ring 3-6 mm | | 1.74 (0.07) | | 1.44 (0.14) | **0.048** |
|  | Ring 6-9 mm | | 2.46 (0.09) | | 1.97 (0.19) | **0.019** |
|  | Ring 9-12 mm | | 3.22 (0.11) | | 2.53 (0.24) | **0.010** |
| Inferior |  |  |  |  |  |  |
|  | Ring 3-6 mm | | 0.44 (0.02) | | 0.38 (0.04) | 0.132 |
|  | Ring 6-9 mm | | 0.62 (0.02) | | 0.51 (0.05) | 0.076 |
|  | Ring 9-12 mm | | 0.74 (0.03) | | 0.61 (0.06) | 0.063 |
| Nasal |  |  |  |  |  |  |
|  | Ring 3-6 mm | | 0.40 (0.02) | | 0.33 (0.04) | 0.120 |
|  | Ring 6-9 mm | | 0.44 (0.02) | | 0.35 (0.05) | 0.111 |
|  | Ring 9-12 mm | | 0.56 (0.03) | | 0.45 (0.06) | 0.120 |
| **CVI (%)** |  |  |  |  |  |  |
| Total |  |  |  |  |  |  |
|  | Circle 0-3 mm | | 0.31 (0.01) | | 0.30 (0.01) | 0.555 |
|  | Circle 0-6 mm | | 0.32 (0.02) | | 0.29 (0.01) | 0.082 |
|  | Circle 0-9 mm | | 0.33 (0.03) | | 0.27 (0.01) | 0.103 |
|  | Circle 0-12 mm | | 0.35 (0.05) | | 0.27 (0.01) | 0.157 |
|  | Ring 3-6 mm | | 0.31 (0.01) | | 0.28 (0.01) | **0.026** |
|  | Ring 6-9 mm | | 0.30 (0.01) | | 0.26 (0.01) | **0.012** |
|  | Ring 9-12 mm | | 0.31 (0.02) | | 0.28 (0.01) | **0.039** |
| Superior |  |  |  |  |  |  |
|  | Ring 3-6 mm | | 0.31 (0.01) | | 0.28 (0.01) | **0.019** |
|  | Ring 6-9 mm | | 0.31 (0.01) | | 0.29 (0.01) | **0.005** |
|  | Ring 9-12 mm | | 0.31 (0.01) | | 0.28 (0.01) | **0.003** |
| Temporal | |  |  |  |  |  |
|  | Ring 3-6 mm | | 0.28 (0.01) | | 0.26 (0.01) | **0.028** |
|  | Ring 6-9 mm | | 0.28 (0.01) | | 0.26 (0.01) | **0.005** |
|  | Ring 9-12 mm | | 0.30 (0.01) | | 0.27 (0.01) | **0.007** |
| Inferior |  |  |  |  |  |  |
|  | Ring 3-6 mm | | 0.31 (0.01) | | 0.29 (0.01) | 0.236 |
|  | Ring 6-9 mm | | 0.30 (0.01) | | 0.28 (0.01) | 0.054 |
|  | Ring 9-12 mm | | 0.31 (0.01) | | 0.29 (0.01) | 0.120 |
| Nasal |  |  |  |  |  |  |
|  | Ring 3-6 mm | | 0.32 (0.01) | | 0.29 (0.01) | **0.015** |
|  | Ring 6-9 mm | | 0.26 (0.01) | | 0.23 (0.01) | **0.003** |
|  | Ring 9-12 mm | | 0.26 (0.01) | | 0.24 (0.01) | 0.133 |

**Abbreviations:** CVI, choroidal vascular index; CVV, choroidal vascular volume; DME, diabetic macular edema; SD, standard deviation.

# Supplementary Table S7. Comparison of retinal and choroidal thickness between eyes with and without DME.

| Region |  | Eyes without DME  (N = 147) | | | Eyes with DME  (N = 44) | | P value |
| --- | --- | --- | --- | --- | --- | --- | --- |
|  |  |  | Mean (SD) | | | Mean (SD) |  |
| **GCC thickness(μm)** | |  |  |  | |  |  |
| Total |  |  |  |  | |  |  |
|  | Circle 0-3 mm | 107.83 (1.47) | | | 127.57 (5.83) | | **0.001** |
|  | Circle 0-6 mm | 110.35 (1.39) | | | 123.82 (3.72) | | **0.001** |
|  | Circle 0-9 mm | 113.80 (1.52) | | | 124.78 (3.08) | | **0.001** |
|  | Circle 0-12 mm | 111.12 (1.52) | | | 119.95 (3.48) | | **0.020** |
|  | Ring 3-6 mm | 111.21 (1.45) | | | 122.56 (3.36) | | **0.002** |
|  | Ring 6-9 mm | 116.59 (1.73) | | | 125.47 (3.18) | | **0.014** |
|  | Ring 9-12 mm | 107.67 (1.98) | | | 113.51 (4.40） | | 0.226 |
| Superior |  |  |  |  | |  |  |
|  | Ring 3-6 mm | 112.88 (1.60) | | | 123.59 (4.57) | | **0.027** |
|  | Ring 6-9 mm | 100.07 (1.57) | | | 111.94 (4.00) | | **0.006** |
|  | Ring 9-12 mm | 29.57 (0.48) | | | 32.25 (1.65） | | **0.013** |
| Temporal |  |  |  |  | |  |  |
|  | Ring 3-6 mm | 95.12 (1.49) | | | 104.70 (2.74) | | **0.002** |
|  | Ring 6-9 mm | 74.64 (1.58) | | | 82.71 (2.51) | | **0.006** |
|  | Ring 9-12 mm | 66.42 (1.48) | | | 71.22 (2.14） | | 0.065 |
| Inferior |  |  |  |  | |  |  |
|  | Ring 3-6 mm | 108.48 (1.52) | | | 119.90 (3.93) | | **0.007** |
|  | Ring 6-9 mm | 104.08 (1.70) | | | 113.11 (4.80) | | 0.076 |
|  | Ring 9-12 mm | 83.03 (1.94) | | | 91.31 (5.21） | | 0.137 |
| Nasal |  |  |  |  | |  |  |
|  | Ring 3-6 mm | 128.09 (1.68) | | | 142.32 (4.89) | | **0.006** |
|  | Ring 6-9 mm | 187.57 (3.40) | | | 194.11 (6.46) | | 0.371 |
|  | Ring 9-12 mm | 188.09 (4.35) | | | 181.44 (8.87） | | 0.501 |
| **RNFL thickness (μm)** | |  |  |  | |  |  |
| Total |  |  |  |  | |  |  |
|  | Circle 0-3 mm | 30.55 (0.79) | | | 39.62 (2.49) | | **0.001** |
|  | Circle 0-6 mm | 46.49 (0.84) | | | 55.73 (2.29) | | **<0.001** |
|  | Circle 0-9 mm | 65.79 (1.14) | | | 73.78 (2.20) | | **0.001** |
|  | Circle 0-12 mm | 71.30 (1.18) | | | 76.90 (2.77) | | 0.063 |
|  | Ring 3-6 mm | 51.80 (0.91) | | | 61.09 (2.32) | | **<0.001** |
|  | Ring 6-9 mm | 81.25 (1.47) | | | 88.24 (2.66) | | **0.021** |
|  | Ring 9-12 mm | 78.50 (1.80) | | | 81.00 (4.02） | | 0.571 |
| Superior |  |  |  |  | |  |  |
|  | Ring 3-6 mm | 55.49 (1.04) | | | 64.52 (3.10) | | **0.006** |
|  | Ring 6-9 mm | 62.90 (1.35) | | | 74.52 (3.52) | | **0.005** |
|  | Ring 9-12 mm | 61.56 (1.72) | | | 68.09 (3.74） | | 0.113 |
| Temporal |  |  |  |  | |  |  |
|  | Ring 3-6 mm | 30.13 (0.90) | | | 37.26 (1.62) | | **<0.001** |
|  | Ring 6-9 mm | 35.28 (1.26) | | | 40.78 (1.80) | | **0.012** |
|  | Ring 9-12 mm | 36.57 (1.22) | | | 39.02 (1.94） | | 0.285 |
| Inferior |  |  |  |  | |  |  |
|  | Ring 3-6 mm | 56.27 (1.00) | | | 64.59 (2.74) | | **0.004** |
|  | Ring 6-9 mm | 70.50 (1.42) | | | 76.13 (3.72) | | 0.158 |
|  | Ring 9-12 mm | 53.78 (1.67) | | | 60.47 (3.52） | | 0.086 |
| Nasal |  |  |  |  | |  |  |
|  | Ring 3-6 mm | 65.23 (1.22) | | | 78.17 (3.56) | | **0.001** |
|  | Ring 6-9 mm | 155.28 (1.47) | | | 161.27 (6.08) | | 0.389 |
|  | Ring 9-12 mm | 161.54 (4.42) | | | 152.30 (9.09） | | 0.361 |
| **GCL+IPL (μm)** |  |  |  |  | |  |  |
| Total |  |  |  |  | |  |  |
|  | Circle 0-3 mm | 77.12 (1.08) | | | 88.01 (3.76) | | **0.005** |
|  | Circle 0-6 mm | 63.74 (0.84) | | | 68.19 (2.05) | | **0.045** |
|  | Circle 0-9 mm | 47.79 (0.61) | | | 51.07 (1.75) | | 0.078 |
|  | Circle 0-12 mm | 39.51 (0.50) | | | 43.18 (1.91) | | 0.063 |
|  | Ring 3-6 mm | 59.29 (0.84) | | | 61.60 (1.82) | | 0.250 |
|  | Ring 6-9 mm | 35.02 (0.51) | | | 37.29 (1.98) | | 0.266 |
|  | Ring 9-12 mm | 28.81 (0.50) | | | 32.65 (2.63） | | 0.152 |
| Superior |  |  |  |  | |  |  |
|  | Ring 3-6 mm | 57.19 (0.85) | | | 59.12 (2.20) | | 0.413 |
|  | Ring 6-9 mm | 35.86 (0.42) | | | 37.44 (1.42) | | 0.286 |
|  | Ring 9-12 mm | 30.70 (0.96) | | | 38.83 (4.92） | | 0.105 |
| Temporal |  |  |  |  | |  |  |
|  | Ring 3-6 mm | 64.99 (1.11) | | | 67.49 (2.15) | | 0.301 |
|  | Ring 6-9 mm | 39.24 (0.64) | | | 41.94 (1.50) | | 0.099 |
|  | Ring 9-12 mm | 29.57 (0.48) | | | 32.25 (1.65） | | 0.118 |
| Inferior |  |  |  |  | |  |  |
|  | Ring 3-6 mm | 52.08 (0.85) | | | 55.32 (1.91) | | 0.120 |
|  | Ring 6-9 mm | 33.18 (0.82) | | | 37.12 (4.31) | | 0.368 |
|  | Ring 9-12 mm | 28.94 (0.92) | | | 30.94 (3.74） | | 0.605 |
| Nasal |  |  |  |  | |  |  |
|  | Ring 3-6 mm | 62.71 (0.90) | | | 64.46 (2.63) | | 0.528 |
|  | Ring 6-9 mm | 31.72 (0.52) | | | 32.90 (1.89) | | 0.546 |
|  | Ring 9-12 mm | 26.03 (0.45) | | | 29.11 (2.30） | | 0.190 |
| **Choroid (μm)** |  |  | | |  | |  |
| Total |  |  | | |  | |  |
|  | Circle 0-3 mm | 351.59 (12.47) | | | 304.22 (25.78) | | 0.098 |
|  | Circle 0-6 mm | 329.97 (11.45) | | | 283.97 (23.06) | | 0.074 |
|  | Circle 0-9 mm | 304.41 (9.96) | | | 259.73 (19.69) | | **0.043** |
|  | Circle 0-12 mm | 286.48 (8.61) | | | 243.50 (16.92) | | **0.024** |
|  | Ring 3-6 mm | 322.78 (11.16) | | | 277.22 (22.23) | | 0.067 |
|  | Ring 6-9 mm | 283.98 (8.91) | | | 240.22 (17.16) | | **0.024** |
|  | Ring 9-12 mm | 263.31 (7.11) | | | 221.43 (13.68） | | **0.007** |
| Superior |  |  | | |  | |  |
|  | Ring 3-6 mm | 343.43 (12.23) | | | 289.30 (21.91) | | 0.310 |
|  | Ring 6-9 mm | 332.54 (10.83) | | | 272.22 (18.43) | | **0.005** |
|  | Ring 9-12 mm | 323.13 (9.19) | | | 256.85 (15.15） | | **<0.001** |
| Temporal |  |  | | |  | |  |
|  | Ring 3-6 mm | 337.56 (10.71) | | | 286.38 (21.24) | | **0.031** |
|  | Ring 6-9 mm | 307.22 (8.82) | | | 262.59 (16.72) | | **0.018** |
|  | Ring 9-12 mm | 285.28 (6.88) | | | 244.77 (13.63） | | **0.008** |
| Inferior |  |  | | |  | |  |
|  | Ring 3-6 mm | 318.42 (10.39) | | | 283.23 (23.03) | | 0.164 |
|  | Ring 6-9 mm | 277.01 (8.12) | | | 242.43 (18.8) | | 0.091 |
|  | Ring 9-12 mm | 243.37 (6.39) | | | 214.82 (14.99） | | 0.080 |
| Nasal |  |  | | |  | |  |
|  | Ring 3-6 mm | 292.00 (12.88) | | | 250.10 (24.96) | | 0.136 |
|  | Ring 6-9 mm | 219.33 (10.26) | | | 183.97 (18.08) | | 0.089 |
|  | Ring 9-12 mm | 201.35 (8.56) | | | 171.67 (15.17） | | 0.088 |

**Abbreviations:** DME, diabetic macular edema; GCC, ganglion cell complex; GCL+IPL, ganglion cell layer plus inner plexiform layer; RNFL, retinal nerve fiber layer; SD, standard deviation.

# Supplementary Table S8. Univariate Cox regression analyses of factors associated with DR progression and DME development.

| Variable (unit) | | DR Progression | | | |  | | DME Development | | | |
| --- | --- | --- | --- | --- | --- | --- | --- | --- | --- | --- | --- |
|  | | HR (95% CI) | | P | |  | | HR (95% CI) | | P | |
| Treatment Group (SUL+INS) | | Ref. | | - | |  | | Ref. | | - | |
|  | |  | |  | |  | |  | |  | |
| SGLT2i + INS | | 0.52 (0.28-0.95) | | **0.034** | |  | | 0.56 (0.19-1.63) | | 0.288 | |
| Age (per year) | | 1.00 (0.97-1.03) | | 0.935 | |  | | 1.00 (0.95-1.05) | | 0.955 | |
| Sex (female) | | Ref. | | - | |  | | Ref. | | - | |
| Male | | 1.33 (0.73-2.40) | | 0.352 | |  | | 1.02 (0.37-2.86) | | 0.965 | |
| Duration of diabetes (per year) | | 1.02 (0.99-1.06) | | 0.240 | |  | | 1.01 (0.94-1.08) | | 0.758 | |
| Body mass index (kg/m²) | | 0.98 (0.86-1.11) | | 0.715 | |  | | 0.91 (0.73-1.14) | | 0.425 | |
| logMAR | | 0.94 (0.43-2.06) | | 0.880 | |  | | 0.47 (0.08-2.79) | | 0.405 | |
| Severity of DR | |  | | **0.017*** | |  | |  | | **0.007*** | |
| Moderate NPDR | | Ref. | | - | |  | | Ref. | | - | |
| Severe NPDR | | 1.77 (0.92-3.41) | | 0.090 | |  | | 1.32 (0.39-4.53) | | 0.658 | |
| PDR | | 2.89 (1.17-7.12) | | 0.021 | |  | | 8.52 (2.22-32.73) | | 0.002 | |
| HbA1c (%) | | 1.29 (1.04-1.60) | | **0.021** | |  | | 1.25 (0.85-1.85) | | 0.255 | |
| Comorbidities | |  | |  | |  | |  | |  | |
| Hypertension (No) | | Ref. | | - | |  | | Ref. | | - | |
| Yes | | 1.20 (0.68-2.12) | | 0.523 | |  | | 1.75 (0.46-6.67) | | 0.414 | |
| Hyperlipidemia (No) | | Ref. | | - | |  | | Ref. | | - | |
| Yes | | 0.86 (0.50-1.49) | | 0.593 | |  | | 1.13 (0.41-3.16) | | 0.810 | |
| DN (No) | | Ref. | | - | |  | | Ref. | | - | |
| Yes | | 2.10 (1.21-3.65) | | **0.008** | |  | | 2.16 (0.78-5.95) | | 0.137 | |
| Smoke | |  | | 0.683* | |  | |  | | 0.708* | |
| Never | | Ref. | | - | |  | | Ref. | | - | |
| Former | | 1.77 (0.86-3.67) | | 0.122 | |  | | 0.68 (0.14-3.15) | | 0.617 | |
| Current | | 0.70 (0.31-1.61) | | 0.406 | |  | | 0.51 (0.11-2.34) | | 0.388 | |
| SS-OCTA Metrics ^a^ | |  | |  | |  | |  | |  | |
| VD of SCP | |  | | **0.020*** | |  | |  | | 0.776* | |
| Q1 (highest) | | Ref. | | - | |  | | Ref. | | - | |
| Q2 | | 1.27 (0.5-3.19) | | 0.615 | |  | | 0.29 (0.06-1.52) | | 0.143 | |
| Q3 | | 1.96 (0.81-4.79) | | 0.138 | |  | | 0.93 (0.28-3.06) | | 0.904 | |
| Q4 (lowest) | | 2.38 (0.99-5.71) | | 0.052 | |  | | 0.56 (0.14-2.36) | | 0.433 | |
| VD of DCP | |  | | **0.026*** | |  | |  | | 0.588* | |
| Q1 (highest) | | Ref. | | - | |  | | Ref. | | - | |
| Q2 | | 1.56 (0.63-3.87) | | 0.338 | |  | | 0.99 (0.22-4.44) | | 0.991 | |
| Q3 | | 2.03 (0.90-4.57) | | 0.088 | |  | | 1.00 (0.25-3.99) | | 0.995 | |
| Q4 (lowest) | | 2.35 (1.06-5.23) | | 0.035 | |  | | 1.47 (0.39-5.49) | | 0.565 | |
| PA of SCP | |  | | 0.195* | |  | |  | | 0.929* | |
| Q1 (highest) | | Ref. | | - | |  | | Ref. | | - | |
| Q2 | | 0.52 (0.22-1.21) | | 0.129 | |  | | 1.13 (0.27-4.76) | | 0.869 | |
| Q3 | | 1.17 (0.55-2.48) | | 0.679 | |  | | 1.32 (0.31-5.51) | | 0.708 | |
| Q4 (lowest) | | 1.21 (0.57-2.56) | | 0.619 | |  | | 1.00 (0.20-4.98) | | 0.997 | |
| PA of DCP | |  | | **0.001*** | |  | |  | | 0.440* | |
| Q1 (highest) | | Ref. | | - | |  | | Ref. | | - | |
| Q2 | | 2.23 (0.79-6.28) | | 0.127 | |  | | 1.96 (0.38-10.14) | | 0.422 | |
| Q3 | | 4.22 (1.57-11.31) | | 0.004 | |  | | 3.62 (0.75-17.45) | | 0.109 | |
| Q4 (lowest) | | 4.18 (1.55-11.29) | | 0.005 | |  | | 1.36 (0.19-9.71) | | 0.756 | |

**Note:** ^a^ SS-OCTA metrics derived from the macula-centered 6 × 6 mm scan were categorized into quartiles and included in the Cox regression analyses.

* P for trend.

**Abbreviations:** CI, confidence interval; DCP, deep capillary plexus; DME, diabetic macular edema; DN, diabetic nephropathy; DR, diabetic retinopathy; HbA1c, glycated hemoglobin; INS, insulin; logMAR, logarithm of the minimum angle of resolution; NPDR, non-proliferative diabetic retinopathy; PA, perfusion area; PDR, proliferative diabetic retinopathy; Ref., reference group; SCP, superficial capillary plexus; SGLT2i, sodium-glucose cotransporter 2 inhibitor; SS-OCTA, swept-source optical coherence tomography angiography; SUL, sulfonylurea; VD, vessel density.

# Supplementary Table S9. Collinearity diagnosis of risk factors for diabetic retinopathy progression.

| Variable ^a^ | Tolerance | VIF |
| --- | --- | --- |
| Treatment Group | 0.911 | 1.097 |
| Age | 0.752 | 1.330 |
| Sex | 0.870 | 1.149 |
| Duration of diabetes | 0.719 | 1.391 |
| HbA1c | 0.934 | 1.071 |
| Severity of DR | 0.871 | 1.148 |
| DN | 0.859 | 1.164 |
| VD of SCP | 0.960 | 1.042 |
| VD of DCP | 0.936 | 1.069 |

**Note:** ^a^ Variables with P < 0.1 in univariate Cox regression analyses were included; demographic factors were retained as baseline covariates in all models.

**Abbreviations:** DCP, deep capillary plexus; DN, diabetic nephropathy; DR, diabetic retinopathy; HbA1c, glycated hemoglobin; SCP, superficial capillary plexus; VD, vessel density; VIF, variance inflation factor.

# Supplementary Table S10. Tests of proportional hazards assumption for Model 3.

| Variable | χ² | DF | P value |
| --- | --- | --- | --- |
| Treatment Group | 0.7665 | 1 | 0.381 |
| Sex | 6.03 | 1 | 0.014 |
| Age | 0.31 | 1 | 0.581 |
| Duration of diabetes | 2.50 | 1 | 0.114 |
| HbA1c | 0.06 | 1 | 0.805 |
| DN | 0.03 | 1 | 0.864 |
| VD of SCP | 0.91 | 3 | 0.824 |
| VD of DCP | 0.63 | 3 | 0.89 |
| Global | 14.03 | 12 | 0.299 |

**Abbreviation**: DCP, deep capillary plexus; DF, degrees of freedom; DN, diabetic nephropathy; HbA1c, glycated hemoglobin; SCP, superficial capillary plexus; VD, vessel density; χ², chi-square.

# Supplementary Table S11. Descriptive statistics for DN and HbA1c before and after multiple imputation.

| Variable | Variable type | Before MI: Missing rate (%) (n) | Before MI: Statistical measures |  | After MI: Missing rate (%) (n) | After MI: Statistical measures |
| --- | --- | --- | --- | --- | --- | --- |
| DN | Dichotomous (0/1) | 2.62 (5) | Range, 0–1 |  | 0.00 (955) | Range, 0–1 |
| HbA1c | Continuous (%) | 5.76 (11) | Mean ± SD,  8.74 ± 1.36 |  | 0.00 (955) | Mean ± SD, 8.72 ± 1.36 |
|  |  |  | Range, 6.0–12.2 |  |  | Range, 5.34–12.2 |

**Abbreviations:**DN, diabetic nephropathy; HbA1c, glycated hemoglobin; MI, multiple imputation; SD, standard deviation.

# Supplementary Table S12. Covariate balance before and after inverse probability of treatment weighting.

| Covariate | Mean unweighted SMD | Mean unweighted SMD |
| --- | --- | --- |
| Age (years) | 0.265 | 0.020 |
| Sex (Male vs Female) | 0.312 | 0.021 |
| Diabetes Duration (years) | 0.036 | 0.071 |
| HbA1c (%) | 0.191 | 0.044 |
| DR Severity  Severe NPDR | 0.347 | 0.007 |
| DR Severity  Moderate NPDR | 0.259 | 0.008 |
| DR Severity  PDR | 0.155 | 0.003 |
| DN (Yes vs No) | 0.243 | 0.170 |
| SCP VD: Q1 | 0.177 | 0.005 |
| SCP VD: Q2 | 0.032 | 0.035 |
| SCP VD: Q3 | 0.058 | 0.017 |
| SCP VD: Q4 | 0.090 | 0.025 |
| DCP VD: Q1 | 0.446 | 0.006 |
| DCP VD: Q2 | 0.123 | 0.004 |
| DCP VD: Q3 | 0.129 | 0.058 |
| DCP VD: Q4 | 0.479 | 0.051 |

**Notes:** SMDs are presented as averages across the five multiply imputed datasets. Smaller absolute SMD values indicate better covariate balance, and values <0.1 were considered indicative of good balance.

**Abbreviations:** DCP, deep capillary plexus; DN, diabetic nephropathy; DR, diabetic retinopathy; IPTW, inverse probability of treatment weighting; NPDR, non-proliferative diabetic retinopathy; PDR, proliferative diabetic retinopathy; SCP, superficial capillary plexus; SMD, standardized mean difference.

# Supplementary Table S13. Comparison of candidate models and bootstrap-based internal validation of the exploratory prediction model for diabetic retinopathy progression.

| Panel A. Comparison of candidate models | | | | | | | |  | |  | |
| --- | --- | --- | --- | --- | --- | --- | --- | --- | --- | --- | --- |
|  | Likelihood ratio test | | |  | C-index | | |  | | AIC | |
|  | χ² | DF | P |  | C-index  (95% CI) | ΔC-index | P for ΔC-index | |  |  |  |
| Model 1 ^a^ | 7.94 | 4 | 0.100 |  | 0.566  (0.468- 0.664) | - | - | |  | 466.1 |  |
| Model 2 ^b^ | 21.26 | 8 | 0.006 |  | 0.651  (0.551- 0.751) | 0.107 | 0.295 | |  | 405.1 |  |
| Model 3 ^c^ | 31.43 | 14 | 0.005 |  | 0.705  (0.597- 0.813) | 0.162 | 0.001 | |  | 406.3 |  |

| Panel B. Bootstrap internal validation of the final model (Model 3) | | | | |
| --- | --- | --- | --- | --- |
| Performance metric | Apparent | Optimism | Corrected | Bootstrap 95% CI |
| C-index | 0.705 | 0.083 | 0.623 | 0.531- 0.711 |
| Calibration slope | 1.000 | 0.422 | 0.578 | 0.259- 0.950 |

**Notes**:

^a^ Model 1 was adjusted for treatment group, age, sex, and diabetes duration.

^b^ Model 2 included Model 1 plus HbA1c, baseline DR severity, and diabetic nephropathy.

^c^ Model 3 included Model 2 plus vessel density in the SCP and DCP (categorical variables).

ΔC-index indicates the absolute difference in C-index compared with Model 1, and P for ΔC-index indicates the corresponding P value. Internal validation was performed using 1000 bootstrap resamples. Bootstrap 95% CIs were derived from the empirical bootstrap distribution.

**Abbreviations:** AIC, Akaike information criterion; CI, confidence interval; C-index, concordance index; DCP, deep capillary plexus; DF, degrees of freedom; DR, diabetic retinopathy; HbA1c, glycated hemoglobin; SCP, superficial capillary plexus; χ², chi-square.

# Supplementary Table S14. Mendelian randomization analysis of the causal association between the SGLT2 target (SLC5A2) and DR.

| ID | Exposure | Outcome | nSNPs | IVW | | MR-Egger | | Weighted median | | Simple mode | | Weighted mode | |
| --- | --- | --- | --- | --- | --- | --- | --- | --- | --- | --- | --- | --- | --- |
|  |  |  |  | OR (95% CI) | *P* | OR (95% CI) | *P* | OR (95% CI) | *P* | OR (95% CI) | *P* | OR (95% CI) | *P* |
| eqtl-a-ENSG00000140675 | SGLT2 | DR | 9 | 1.21 (1.05 - 1.39) | 8.92E-03 | 1.43 (0.69 - 2.98) | 3.73E-01 | 1.16 (0.97 -1.38) | 1.17E-01 | 1.15 (0.90 - 1.47) | 2.89E-01 | 1.15 (0.91- 1.45) | 2.77E-01 |

**Abbreviations:** CI, confidence interval; DR, diabetic retinopathy; IVW, inverse-variance weighted; MR, Mendelian randomization; MR-Egger, Mendelian randomization Egger regression; nSNPs, number of single-nucleotide polymorphisms; OR, odds ratio; SGLT2, sodium-glucose cotransporter 2; SLC5A2, solute carrier family 5 member 2.

# Supplementary Table S15. Heterogeneity and horizontal pleiotropy in Mendelian randomization analyses of the SGLT2 target (SLC5A2) and DR.

| Exposure | Outcome | Method | nSNPs | Heterogeneity | |  | Horizontal Pleiotropy | | |
| --- | --- | --- | --- | --- | --- | --- | --- | --- | --- |
|  |  |  |  | Q | DF | P value | MR-Egger | |  |
|  |  |  |  |  |  |  | Egger Intercept | SE | P value |
| SLC5A2 | DR | MR-Egger | 9 | 1.69 | 7 | 0.975 | -0.013 | 0.027 | 0.660 |
| SLC5A2 | DR | IVW | 9 | 1.91 | 8 | 0.984 |  |  |  |

**Abbreviations:** DF, degrees of freedom; DR, diabetic retinopathy; IVW, inverse-variance weighted; MR, Mendelian randomization; MR-Egger, Mendelian randomization Egger regression; nSNPs, number of single-nucleotide polymorphisms; SE, standard error; SGLT2, sodium-glucose cotransporter 2; SLC5A2, solute carrier family 5 member 2.

# Supplementary Table S16. Mediating effects of the SGLT2 target (SLC5A2) on diabetic retinopathy via plasma proteins and circulating metabolites.

| Mediator | Category |  | Beta (SE), P value | |  | Mediating effect | | | |  |
| --- | --- | --- | --- | --- | --- | --- | --- | --- | --- | --- |
|  |  |  | Exposure-mediator | Mediator-outcome |  | Beta (SE) | 95% CI | P value | FDR-corrected P |  |
| SHANK3 | Plasma Proteins |  | 0.183 (0.043), 2.32E-05 | -0.075 (0.032), 1.78E-02 | -0.014 (0.007) | | (0.972, 0.998) | 4.30E-02 | 1.28E-01 |  |
| TIMP3 | Plasma Proteins |  | 0.153 (0.043), 3.08E-04 | 0.092 (0.028), 8.80E-04 | 0.014 (0.006) | | (1.004, 1.028) | 1.66E-02 | 8.20E-02 |  |
| AGER | Plasma Proteins |  | -0.153 (0.045), 7.03E-04 | -0.499 (0.147), 6.95E-04 | 0.076 (0.033) | | (1.023, 1.161) | 1.93E-02 | 8.20E-02 |  |
| MICB | Plasma Proteins |  | 0.165 (0.039), 2.87E-05 | -0.256 (0.048), 1.11E-07 | -0.042 (0.013) | | (0.932, 0.981) | 1.18E-03 | 2.01E-02 |  |
| STIM1 | Plasma Proteins |  | 0.325 (0.042), 1.07E-14 | -0.319 (0.126), 1.15E-02 | -0.103 (0.043) | | (0.825, 0.979) | 1.75E-02 | 8.20E-02 |  |
| Stearoylcarnitine levels | Metabolites |  | 0.589 (0.082), 9.08E-13 | -0.127 (0.04), 1.45E-03 | -0.075 (0.026) | | (0.880, 0.976) | 4.00E-03 | 9.47E-02 |  |
| Branched-chain, straight-chain, or cyclopropyl 10:1 fatty acid (1) levels | Metabolites |  | 0.518 (0.081), 1.33E-10 | -0.07 (0.025), 5.57E-03 | -0.036 (0.014) | | (0.937, 0.992) | 1.20E-02 | 9.71E-02 |  |
| Glycosyl-N-(2-hydroxynervonoyl)-sphingosine (d18:1/24:1(2OH)) levels | Metabolites |  | -0.228 (0.079), 3.94E-03 | 0.152 (0.038), 5.33E-05 | -0.035 (0.015) | | (0.938, 0.995) | 2.10E-02 | 9.71E-02 |  |
| Octanoylcarnitine (c8) levels | Metabolites |  | 0.295 (0.081), 2.69E-04 | 0.092 (0.031), 3.21E-03 | 0.027 (0.012) | | (1.003, 1.052) | 2.50E-02 | 9.71E-02 |  |
| Cysteine s-sulfate levels | Metabolites |  | 0.406 (0.082), 6.88E-07 | -0.11 (0.044), 1.15E-02 | -0.045 (0.02) | | (0.919, 0.995) | 2.70E-02 | 9.71E-02 |  |
| Cholate to bilirubin (Z, Z) ratio | Metabolites |  | -0.436 (0.087), 5.61E-07 | 0.082 (0.034), 1.47E-02 | -0.036 (0.016) | | (0.934, 0.997) | 3.00E-02 | 9.71E-02 |  |
| Phytanate levels | Metabolites |  | 0.369 (0.083), 8.67E-06 | 0.09 (0.036), 1.18E-02 | 0.033 (0.015) | | (1.003, 1.066) | 3.10E-02 | 9.71E-02 |  |
| Malonylcarnitine levels | Metabolites |  | 0.329 (0.096), 6.14E-04 | 0.095 (0.033), 4.00E-03 | 0.031 (0.015) | | (1.003, 1.062) | 3.20E-02 | 9.71E-02 |  |
| Phosphate to acetoacetate ratio | Metabolites |  | -0.321 (0.092), 4.90E-04 | 0.085 (0.033), 9.43E-03 | -0.027 (0.013) | | (0.948, 0.999) | 4.30E-02 | 9.47E-02 |  |

**Abbreviations:** CI, confidence interval; DR, diabetic retinopathy; FDR, false discovery rate; SE, standard error; SGLT2, sodium-glucose cotransporter 2; SLC5A2, solute carrier family 5 member 2.

# Supplementary Table S17. Heterogeneity and horizontal pleiotropy in two-step Mendelian randomization mediation analyses of the SGLT2 target (SLC5A2) and DR.

| Mediator | Method | nSNPs | |  | Heterogeneity  (Q, P value) ^a^ | |  | Horizontal Pleiotropy (Egger intercept, P value) ^b^ | |
| --- | --- | --- | --- | --- | --- | --- | --- | --- | --- |
|  |  | exp-med | med-out |  | exp-med | med-out |  | exp-med | med-out |
|  |  |  |  |  |  |  |  |  |  |
| SHANK3 | MR-Egger | 9 | 5 |  | 0.61, 9.99E-01 | 0.99, 8.03E-01 | | -0.005, 7.73E-01 | 0.018, 3.35E-01 |
| SHANK3 | IVW | 9 | 5 |  | 0.7, 1.00E+00 | 2.31, 6.80E-01 | |  |  |
| TIMP3 | MR-Egger | 9 | 8 |  | 2.36, 9.37E-01 | 2.52, 8.66E-01 | | -0.006, 7.40E-01 | -0.014, 3.45E-01 |
| TIMP3 | IVW | 9 | 8 |  | 2.48, 9.63E-01 | 3.57, 8.28E-01 | |  |  |
| AGER | MR-Egger | 9 | 2 |  | 2.49, 9.28E-01 | - | | -0.041, 4.19E-02 | - |
| AGER | IVW | 9 | 2 |  | 8.67, 3.71E-01 | 10.54, 1.17E-03 | |  |  |
| MICB | MR-Egger | 9 | 6 |  | 1.11, 9.93E-01 | 13.13, 1.07E-02 | | -0.020, 2.19E-01 | 0.071, 4.04E-01 |
| MICB | IVW | 9 | 6 |  | 2.94, 9.38E-01 | 15.98, 6.91E-03 | |  |  |
| STIM1 | MR-Egger | 9 | 2 |  | 0.6, 9.99E-01 | - | | -0.005, 7.61E-01 | - |
| STIM1 | IVW | 9 | 2 |  | 0.7, 1.00E+00 | 0.33, 5.67E-01 | |  |  |
| Stearoylcarnitine levels | MR-Egger | 9 | 22 |  | 1.24, 9.90E-01 | 15.09, 7.71E-01 | | -0.009, 7.82E-01 | 0.016, 2.09E-01 |
| Stearoylcarnitine levels | IVW | 9 | 22 |  | 1.32, 9.95E-01 | 16.77, 7.25E-01 | |  |  |
| Branched-chain, straight-chain, or cyclopropyl 10:1 fatty acid (1) levels | MR-Egger | 9 | 18 |  | 1.56, 9.80E-01 | 21, 1.79E-01 | | -0.005, 8.79E-01 | 0.001, 9.40E-01 |
| Branched-chain, straight-chain, or cyclopropyl 10:1 fatty acid (1) levels | IVW | 9 | 18 |  | 1.59, 9.91E-01 | 21, 2.26E-01 | |  |  |
| Glycosyl-N-(2-hydroxynervonoyl)-sphingosine (d18:1/24:1(2OH)) levels | MR-Egger | 9 | 26 |  | 3.31, 8.55E-01 | 27.22, 2.94E-01 | | 0.034, 2.97E-01 | -0.009, 3.69E-01 |
| Glycosyl-N-(2-hydroxynervonoyl)-sphingosine (d18:1/24:1(2OH)) levels | IVW | 9 | 26 |  | 4.58, 8.01E-01 | 28.17, 3.00E-01 | |  |  |
| Octanoylcarnitine (c8) levels | MR-Egger | 9 | 33 |  | 0.37, 1.00E+00 | 23.59, 8.27E-01 | | -0.020, 5.34E-01 | 0.004, 6.53E-01 |
| Octanoylcarnitine (c8) levels | IVW | 9 | 33 |  | 0.8, 9.99E-01 | 23.8, 8.52E-01 | |  |  |
| Cysteine s-sulfate levels | MR-Egger | 9 | 17 |  | 0.37, 1.00E+00 | 15.15, 4.41E-01 | | -0.009, 7.72E-01 | 0.006, 6.01E-01 |
| Cysteine s-sulfate levels | IVW | 9 | 17 |  | 0.8, 9.99E-01 | 15.44, 4.93E-01 | |  |  |
| Cholate to bilirubin (Z, Z) ratio | MR-Egger | 9 | 26 |  | 0.34, 1.00E+00 | 12.87, 9.68E-01 | | -0.004, 9.00E-01 | 0, 9.68E-01 |
| Cholate to bilirubin (Z, Z) ratio | IVW | 9 | 26 |  | 0.36, 1.00E+00 | 12.87, 9.78E-01 | |  |  |
| Phytanate levels | MR-Egger | 9 | 22 |  | 1.59, 9.79E-01 | 12.15, 9.11E-01 | | 0.009, 7.83E-01 | 0.009, 3.44E-01 |
| Phytanate levels | IVW | 9 | 22 |  | 1.67, 9.89E-01 | 13.09, 9.06E-01 | |  |  |
| Malonylcarnitine levels | MR-Egger | 9 | 21 |  | 1.38, 9.86E-01 | 19.66, 4.15E-01 | | -0.013, 7.37E-01 | -0.006, 5.71E-01 |
| Malonylcarnitine levels | IVW | 9 | 21 |  | 1.51, 9.93E-01 | 20, 4.58E-01 | |  |  |
| Phosphate to acetoacetate ratio | MR-Egger | 9 | 25 |  | 1.79, 9.70E-01 | 24.79, 3.61E-01 | | 0.011, 7.56E-01 | -0.005, 5.66E-01 |
| Phosphate to acetoacetate ratio | IVW | 9 | 25 |  | 1.9, 9.84E-01 | 25.16, 3.97E-01 | |  |  |

**Notes:** All analyses used the SGLT2 target (SLC5A2) as the exposure and DR as the outcome. The exp–med denotes the exposure–mediator path, and med–out denotes the mediator–outcome path.

^a^ Heterogeneity is presented as Q (DF, P) for the exposure–mediator and mediator–outcome paths, respectively.

^b^ Horizontal pleiotropy is presented as the MR-Egger intercept and its corresponding P value for the exposure–mediator and mediator–outcome paths, respectively. A dash (–) indicates unavailable data for the mediator–outcome path.

**Abbreviations:** DF, degrees of freedom; DR, diabetic retinopathy; exp–med, exposure–mediator path; IVW, inverse-variance weighted; med–out, mediator–outcome path; MR, Mendelian randomization; Q, Cochran’s Q statistic; SGLT2, sodium-glucose cotransporter 2; SLC5A2, solute carrier family 5 member 2.

|  |
| --- |
